# Bayesian Umbrella Quadrature Automates Free-Energy Calculations Across Diverse Molecular Systems and Processes

**DOI:** 10.1021/acs.jctc.6c00120

**Published:** 2026-06-11

**Authors:** Eline K. Kempkes, Alberto Pérez de Alba Ortíz

**Affiliations:** Computational Soft Matter Lab, Computational Chemistry Group and Computational Science Lab, Van ’t Hoff Institute for Molecular Sciences and Informatics Institute, 1234 University of Amsterdam, Science Park 904, 1098 XH Amsterdam, The Netherlands

## Abstract

Biased sampling in molecular dynamics simulations overcomes time scale limitations and delivers free-energy landscapes, essential to understand complex atomistic phenomena. However, when applied across diverse systems and processes, biasing protocols often require time- and resource-consuming fine-tuning. In search of robustness, we automate a prominent biasing method, Umbrella Sampling, by leveraging the Umbrella Integration formalism for free-energy calculation. To estimate the value of an integral, i.e., the free energy, our Bayesian Umbrella Quadrature (BUQ) method iteratively selects gradient samples, i.e., bias locations, that most reduce the posterior integral variance based on a noise-tolerant Gaussian process model, which also effectively interpolates between samples. We validate the method for a conformational change in a small peptide, a water-to-ice phase transition, and a substitution chemical reaction; demonstrating that BUQ provides an automated free-energy calculation framework that is accurate and robust across fundamentally different systems and processes. To ease adoption, we interface BUQ with widespread simulation packages and share hyperparametrization guidelines.

## Graphical abstract

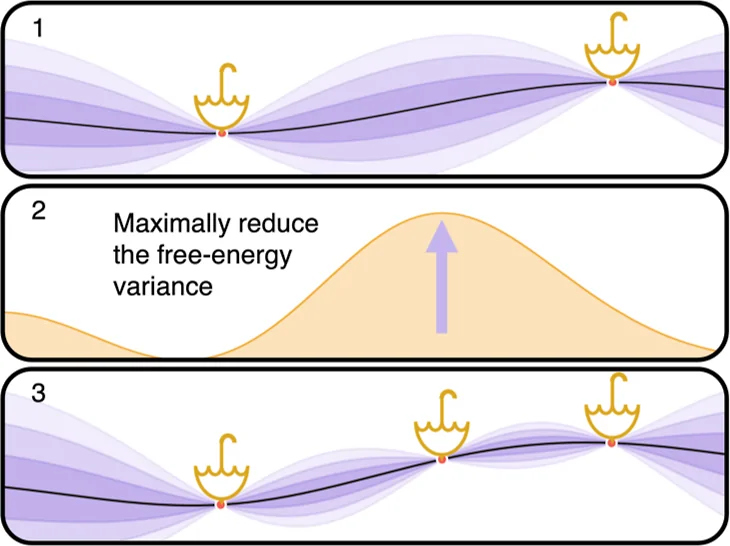

## Introduction

Accurate calculation of free-energy landscapes is central to understanding and predicting chemical reactions and conformational changes in molecules as well as phase transitions in materials. These landscapes are projected onto key degrees of freedom for the system and process at hand, i.e., reaction coordinates, collective variables (CVs), or order parameters, which we will refer to as CVs without loss of generality. Free energies quantify the occurrences, i.e., probabilities, of metastable states and the transition rates and mechanisms between them. Since transitions of interest often occur on time scales inaccessible to standard molecular dynamics (MD) simulations,^1^ enhanced sampling methods have been introduced.^2,3^ Among these, biasing approaches such as steered molecular dynamics^4,5^ or metadynamics^6,7^ accelerate sampling by applying external potentials that guide the system toward infrequently sampled regions. More recently, on-the-fly probability enhanced sampling (OPES) has been proposed, which constructs an adaptive bias to drive the system toward a target distribution in CV space.^8^ One of the most well-established biasing methods is Umbrella Sampling (US),^9^ with over 400 citations per year since 2019 and growing, according to Google Scholar. The main idea of US is to use a series of biased simulations, or windows, each restraining the system around a specific region of the *d*-dimensional CV-space.^10^ In standard US, a typical biasing potential for the *i*th window, acting on the *j*th CV, is defined as

1 $$V_{bias}^{\left(i\right)}\left(s_{j}\right)=\frac{1}{2}\kappa_{j}^{\left(i\right)}{\left(s_{j}-{ŝ}_{j}^{\left(i\right)}\right)}^{2}$$

For simplicity, it is assumed that the CVs, **s** = (*s*_1_, ..., *s*_*d*_), are independent, however, this can be extended to dependent CVs^10,11^ (see Methods). ${ŝ}_{j}^{\left(i\right)}$ denotes the bias center for window *i*, for CV *s*_*j*_, with a harmonic force constant κ_*j*_^(*i*)^. Using multiple biased simulations at different values of ${ŝ}^{\left(i\right)}$ , relevant regions of CV-space can be explored. The free-energy landscape can be reconstructed using the Weighted Histogram Analysis Method (WHAM), which works by combining bias-corrected histograms from multiple windows.^12^ US data can also be processed by the Multistate Bennett Acceptance Ratio (MBAR) method, which works by statistically reweighting samples from multiple thermodynamic states to estimate free energies.^13^ A limitation of both approaches is that the sampled distributions from adjacent umbrellas must overlap sufficiently, and in WHAM, the choice of bin size directly influences the estimated distributions. An alternative is Umbrella Integration (UI),^14^ where we use the negative of the ensemble-averaged biasing force to approximate the free-energy gradient for window *i*

2 $$\frac{\partial A{\left(s_{j}\right)}^{\left(i\right)}}{\partial s_{j}}\approx -\frac{\partial V_{bias}{\left(s_{j}\right)}^{\left(i\right)}}{\partial s_{j}}=-\kappa_{j}^{\left(i\right)}\left({\mathrm{s̅}}_{j}^{\left(i\right)}-{ŝ}_{j}^{\left(i\right)}\right)$$

Afterward, numerical integration can be used to recover the full free-energy surface. Note that this approximation requires a unimodal distribution of the ensemble-averaged biasing force at each window. Here, as is done frequently in literature, we refer to the resulting potential of mean force (PMF) as a free energy, but we note that the exact relation between the two depends on the selection of CVs and contribution of the Jacobian.^15^ Compared to WHAM and MBAR, UI substantially reduces errors associated with histogram discretization and does not require window overlap.^14,16^ This latter property is particularly useful in practice, because it makes UI much less sensitive to the precise value of the force constant, κ, which no longer has to be tuned to enforce overlap between neighboring umbrellas. This makes UI easy to automate, requiring only averaging the biasing force and verifying its unimodality. A disadvantage is that the gradient of the free energy might change in an unsampled region, resulting in an inaccurate free-energy calculation.

The challenge in UI, US and restrained MD methods in general lies in the careful tuning of the parameters, e.g., the number, strength (i.e., κ_*j*_^(*i*)^), and positions $\left({ŝ}^{\left(i\right)}\right)$ of the biasing potentials. This can be challenging for complex systems, especially those involving more than one CV. Moreover, for different processes—e.g., protonation reactions, ligand dissociation, homogeneous nucleation—these settings can be extremely different, since they correspond to fundamentally different phenomena and interactions—e.g., covalent bonds, hydrogen bonds, van der Waals forces, electrostatic repulsion or attraction, etc. This makes the tuning process typically system-specific and highly nontrivial, since unknown barrier heights, metastable valleys and other landscape features must be resolved.^16,17^ This prevents generalization and performance optimization across diverse systems, since poorly chosen biasing parameters can lead to undersampled regions and to wasted computational efforts. Even when dealing with similar systems, small modifications—e.g., changes in pH, the presence of an ion, or a change in temperature—can reshape the free-energy landscape in such way that a former biasing protocol becomes ineffective.

In other words, UI and US protocols are not transferable and therefore can become inefficient and inaccurate when applied across diverse systems. To overcome these limitations, several adaptive schemes have been proposed. Early approaches introduced feedback-based optimization of biasing potentials and window placement to enhance sampling efficiency.^18–22^ More recent developments have incorporated self-learning frameworks that adjust these parameters on the fly,^23,24^ and used machine-learning-based approaches to further generalize this idea.^25,26^ Other prominent adaptive approaches include the Adaptive Biasing Force method family,^27–30^ which constructs a bias from the running estimate of the mean force, and Temperature Accelerated Molecular Dynamics,^31,32^ which uses an elevated fictitious temperature on the CVs to accelerate exploration. Recently, Gaussian process regression has also been successfully applied to reconstruct free-energy surfaces from US and other simulation data, enabling smooth free-energy surface estimation with quantified uncertainty.^33–35^ Related to this, Bayesian inference and related probabilistic approaches have been successfully applied to free-energy estimation, structure refinement, and parameter optimization in molecular systems. Bayesian methods allow for principled treatment of uncertainties, heterogeneous states, and noisy or sparse data. Representative examples include extracting free energies or transition rates from cryogenic electron microscopy data,^36^ path sampling data,^37^ equilibrium and nonequilibrium data,^38–40^ etc.

Inspired by the rise of successful Bayesian approaches, here we propose a Bayesian Umbrella Quadrature (BUQ) framework as a general and automated solution for UI-based free-energy calculations. Bayesian Quadrature (BQ) is an established method for estimating the value of integrals based on noisy samples of the gradient, leveraging Bayesian inference.^41,42^ To our knowledge, this is the first time BQ is integrated into an enhanced sampling MD pipeline. BUQ integrates BQ in a UI framework, where we estimate the value of an integral, i.e., the free-energy landscape, based on iteratively selecting samples of its gradient, i.e., the negative of the force exerted by the umbrellas. BQ uses a Gaussian process (GP), i.e., a noise-tolerant probabilistic model, to estimate the value of the gradient, which can be numerically integrated afterward. Figure 1 illustrates the iterative process. Starting from either prior information about the free-energy gradient or from a few initial gradient samples, BUQ determines the next most informative point to sample the gradient, i.e., the location of the next bias potential, ${ŝ}^{\left(i\right)}$ . Here, the definition of “most informative” is given by an acquisition function, which can be focused on reducing the overall uncertainty of the free energy, i.e., Integral Variance Reduction (IVR), or steered to search for free-energy minima or maxima. This iterative process is repeated such that the integral of the GP gradient surrogate converges to a stable estimate of the free-energy surface (see section Methods).

**Figure 1:**
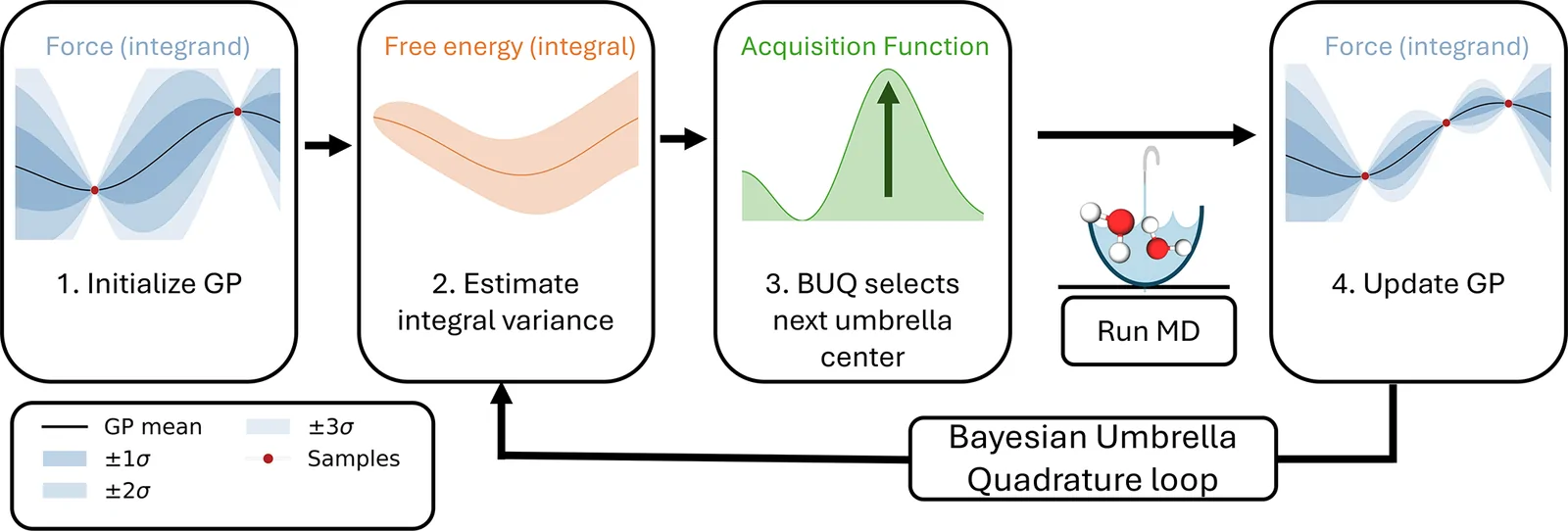
Schematic representation of the Bayesian Umbrella Quadrature (BUQ) loop applied to free-energy calculations. (1) The Gaussian process (GP) gradient model is initialized considering a variance σ and a few initial gradient samples from umbrella-biased molecular dynamics (MD) simulations. (2) Based on the GP gradient model, the variance of the integral, i.e., the free energy, is estimated. (3) Using an acquisition function, BUQ selects the next umbrella center at which sampling the gradient will maximally reduce the variance of the free-energy estimate. (4) A new biased MD simulation is run at the proposed umbrella center, generating a new gradient sample. This new observation is incorporated into the GP model, and the loop is repeated until convergence. This adaptive strategy allows BUQ to prioritize the most informative sampling regions, reducing the computational cost compared to standard grid-based umbrella integration.

The two main strengths of BUQ are (1) the GP interpolates the gradient, providing both an accurate estimate and an associated uncertainty, and (2) the acquisition function guides sampling toward the most informative regions, thereby reducing overall computational effort. This directly addresses the limitations of UI, reducing the risk of undersampling key regions and improving the interpolation of the gradient between samples. An additional strength of BUQ is its ability to integrate existing knowledge, e.g., by integrating data from previous US runs or an initial guess into the GP.

We demonstrate the generality and efficiency of our BUQ method on three diverse benchmark problems of varying nature. First, we benchmark the performance of BUQ against standard UI at calculating the conformational free energy of alanine dipeptide in vacuo projected onto the ϕ and ψ dihedral angles, showing BUQ converges 2.6 times as fast as standard UI, and 1.2 times as fast as UI enhanced with a GP interpolation. We also show a comparable performance to OPES.^8^ Next, to show the robustness of BUQ when handling a collective process, we calculate the nucleation free-energy profile of the phase transition from water to ice Ih, observing a 2.8× speedup considering MD timesteps with respect to variationally enhanced sampling (VES).^43^ Finally, we tackle a different challenge: predicting the barrier of an S_N_2 chemical reaction, where bond breaking introduces fundamentally different interactions, and thus a different free-energy landscape. With these three examples, we show that one of the key strengths of BUQ lies in its robust automation; BUQ can be applied across a wide range of systems and processes, under diverse conditions. Compared to other enhanced sampling methods such as metadynamics or OPES, where crucial hyperparameters (e.g., Gaussian widths and heights, deposition frequency, or barrier parameters) must be chosen before the simulation and suboptimal choices can lead to inefficient sampling or inaccurate free-energy estimates, BUQ offers an important practical advantage. In BUQ, the most critical hyperparameters of the Gaussian process (such as the kernel and its length scale) can be modified a posteriori, allowing the same biased trajectories to be reinterpreted without rerunning MD. This substantially reduces the need for extensive presimulation fine-tuning. Finally, the performance of BUQ does not depend that much on the exact settings of these hyperparameters. To ease the adoption of BUQ, we provide an open-source implementation interfaced with well-established packages for enhanced sampling—i.e., PLUMED^44,45^ —and for MD of different systems; GROMACS,^46^ LAMMPS^47^ and ASE.^48^ All of this considered, BUQ promises a more universal and automated approach to ever more relevant free-energy calculations of molecules and materials. In the present work, we introduce and validate BUQ in the one- and two-dimensional CV regime, which is by far the most common setting in practice:^49–51^ the vast majority of free-energy calculations, including those for larger molecular systems, such as mini-proteins, are projected onto one or two CVs, which may combine different degrees of freedom by using key or global descriptors, distances from reference configurations or machine learning.^52–54^ We note that the selection of appropriate CVs for high-dimensional systems is a general challenge shared by all biased sampling methods and is not specifically addressed here.

## Methods

We begin this section by outlining the standard Umbrella Integration (UI) approach. Next, we discuss the theory of Bayesian Quadrature (BQ) in the context of free-energy estimation, focusing on its two core components: Gaussian processes (GP) and acquisition functions. Finally, we describe the integration of UI in BQ, i.e., Bayesian Umbrella Quadrature (BUQ).

### Umbrella Integration

Umbrella sampling (US) probes free-energy landscapes projected onto a set of key descriptive degrees of freedom—i.e., collective variables (CVs), **s** = (*s*_1_, ..., *s*_*d*_)—using harmonic bias potentials. A typical bias at window *i* for multiple CVs takes the form^10,11^

3 $$V_{bias}^{\left(i\right)}\left(s\right)=\frac{1}{2}{\left(s-{ŝ}^{\left(i\right)}\right)}^{†}K\left(s-{ŝ}^{\left(i\right)}\right)$$

where ${ŝ}^{\left(i\right)}$ denotes the bias center for window *i*, and K denotes the matrix of umbrella force constants. Using multiple biased simulations at different values of ${ŝ}^{\left(i\right)}$ , relevant regions of CV-space can be explored. Multiple biased simulations are carried out in a number of windows. In each biased simulation, an averaged biasing force can be calculated based on the average CV vector in window *i*. Given large enough force constants and unimodal force distributions, each of these average biasing forces can be used to approximate the negative gradient of the underlying free energy at the bias center. The use of the covariance can be avoided by truncation of the Jacobian terms.^11^ This yields the approximation

4 $$\nabla A{\left(s\right)}^{\left(i\right)}{|}_{s={ŝ}^{\left(i\right)}}\approx -K\left(\left\langle s^{\left(i\right)}\right\rangle -{ŝ}^{\left(i\right)}\right)$$

In practice, one collects these gradient estimates from all windows and numerically integrates them to recover the free-energy surface *A*(**s**). It is important to note that, unlike in conventional US combined with WHAM or MBAR, UI does not require overlap between adjacent windows. As a result, the restraining constants κ_*j*_^(*i*)^, which in US must be carefully tuned to balance stability and sufficient overlap, play a less critical role here. In UI, they mainly need to satisfy two conditions: (i) they should not be so large as to produce unphysical distortions or numerical instabilities due to high forces, but (ii) they should be sufficiently strong to confine the CV fluctuations within each window so that the biased CV distribution is unimodal. Under these conditions, the ensemble-averaged biasing force provides a reliable local estimator of the free-energy gradient at the bias center. Both the absence of unphysical distortions and the unimodality of the biased distributions can be verified a posteriori, for example by inspecting structural observables and histograms of the CVs or biasing forces. Thus, by satisfying feasible and verifiable criteria, UI provides an effective way to compute ∇*A* from biased simulations, after which numerical integration yields the free-energy surface *A*(**s**).

### Bayesian Quadrature

Bayesian quadrature (BQ) treats the integral of an expensive function as a Bayesian inference problem. Specifically, it does this by modeling the integrand with a Gaussian process (GP), and refining this surrogate through sequentially selected evaluations of the gradient.^41^ In our application, Bayesian Umbrella Quadrature (BUQ), we aim to compute the free energy with respect to a reference, *A*(**s**_0_), which can be written formally as a path integral of the mean biasing force

$$A\left(s\right)=A\left(s_{0}\right)+\int_{s_{0}}^{s}-F\left(s^{\prime }\right)\cdot ds^{\prime }$$

where −**F**(**s**) is the negative mean force, i.e., the integrand. For simplicity, we set *A*(**s**_0_) = 0. We model the unknown integrand −**F**(**s**) with a GP prior

5 $$-F\left(s\right)∼GP\left(m\left(s\right),K\left(s,s^{\prime }\right)\right)$$

where **m** is the prior mean, often taken equal to zero, and **K** is a matrix-valued covariance kernel. In the independent-CV case, one can use independent GPs for each force component. We use standard stationary kernels—e.g. Radial Basis Function (RBF) or Matérn-ν kernels^55^—to encode smoothness: the RBF kernel implies an infinitely differentiable integrand, whereas Matérn kernels allow for tunable roughness—e.g. Matérn-1/2 permits nondifferentiable, rough functions, while Matérn-5/2 yields a smoother function. For a more detailed discussion, see the Supporting Information. This surrogate GP is then updated with *n* initial samples $D={\left\{(s_{i},-F\left(s_{i}\right))\right\}}_{i=1}^{n}$ from biased MD runs. Each output dimension, i.e., CV, *j* has its own mean *m*_D_(*s*_*j*_) and covariance *k*_D_(*s*_*j*_,*s*_*j*_^′^), given by

6 $$m_{D}\left(s_{j}\right)=m\left(s_{j}\right)+k\left(s_{j},S\right){\left[K\left(S,S\right)\right]}^{-1}\left(y-m\left(S\right)\right)$$

7 $$k_{D}\left(s_{j},s_{j}^{\prime }\right)=k\left(s_{j},s_{j}^{\prime }\right)-k\left(s_{j},S\right){\left[K\left(S,S\right)\right]}^{-1}k\left(S,s_{j}^{\prime }\right)$$

where *S* = [*s*_*j*_1__,...,*s*_*j*_*n*__], i.e., samples of *s*_*j*_, *y* = [−*F*(*s*_*j*_1__),...,–*F*(*s*_*j*_*n*__)]^Τ^, and *k* is the scalar kernel for each component.

Once the posterior is obtained, we can integrate the surrogate analytically to estimate the free energy. In particular, the expected value of the integral is obtained by integrating the GP posterior mean

8 $$E\left[A\right]=\int_{l_{1}}^{u_{1}}\cdot \cdot \cdot \int_{l_{d}}^{u_{d}}m_{D}\left(s_{1},...,s_{d}\right)\;ds_{1}\cdot \cdot \cdot ds_{d}$$

We integrate over finite ranges of the CVs (with lower bounds *l*_1_, ..., *l*_*d*_, and upper bounds *u*_1_, ..., *u*_*d*_). The posterior integral variance of CV *s*_*j*_ can be obtained by integrating the covariance

9 $$V\left[A\right]=\iint k_{D}\left(s_{j},s_{j}^{\prime }\right)\;ds_{j}\;ds_{j}^{\prime }$$

Thus, we model the force function by a GP, giving both a point estimate and uncertainty for *A*. We improve efficiency by actively selecting new sampling points via an acquisition function. A common choice in BQ is the integral-variance-reduction (IVR) criterion, which quantifies how much the posterior variance of the integral would drop by sampling at a candidate point.^56^ Formally, if Vn is the current variance of *A*, then the IVR score for a new point **s** is

10 $$a_{IVR}\left(s\right)=Vn-V\left[A|D\cup s\right]$$

i.e., the reduction in uncertainty by sampling this point. One can also express this as a normalized squared-correlation ρ^2^(**s**) ∈ [0, 1] between the integrand at **s** and the integral. To bias sampling toward low-free-energy regions, we combine IVR with an exploitation term based on the GP predictive mean. Let *a*_FES_(**s**) be the (normalized) GP estimate of *A*(**s**) based on *D*, then we define

11 $$a_{total}\left(s\right)=-\lambda a_{FES}\left(s\right)+\left(1-\lambda \right)a_{IVR}\left(s\right)$$

where λ ∈ [0, 1] balances exploration (variance reduction) against exploitation (favoring lower predicted *A*). This hybrid strategy is analogous to combined exploration-exploitation in Bayesian optimization. For a more detailed discussion, we refer to the Supporting Information.

At each iteration, we maximize the chosen acquisition *a*_total_(**s**) over **s** to select the next sample point **s**_new_. We then perform a restrained MD (US) simulation at **s**_new_ to measure the new mean force −**F**(**s**_new_), add this to the data set *D*, and update the GP to obtain a new posterior mean, i.e., free-energy gradient prediction. In summary, the iterative active BUQ procedure is

1. Initialization: Choose an initial set of ñ samples in CV-space and run biased MD at each of them to obtain forces, $D_{initial}={\left(s_{i},-F\left(s_{i}\right)\right)}_{i=1}^{ñ}$ . Fit a GP (kernel and hyperparameters) to this data.
2. Inference: Compute the GP posterior mean **m**_*D*_(**s**) and covariance **K**_*D*_(**s**, **s**′), then integrate to get E[A] and V[A] . Evaluate the acquisition *a*(**s**) (e.g., IVR or combined).
3. Acquisition: Select the next point **s**_new_ = arg max_**s**_ *a*(**s**). Run a new restrained MD at **s**_new_ to obtain −**F**(**s**_new_).
4. Update: Augment the data set with (**s**_new_, −**F**(**s**_new_)), refit the GP, and repeat steps 2–3 until convergence (e.g., variance is small or landscape does not change significantly).

This loop systematically leverages both the physical insight of UI (averaged biasing forces as approximations to free-energy gradients) and the sample-efficiency of Bayesian quadrature to accelerate free-energy estimation.

## Results

### Conformational Change in Alanine Dipeptide

To demonstrate the performance of our BUQ framework, we start with a conformational transition in a biomolecular system traditionally used to illustrate rare-event sampling, namely, an alanine dipeptide molecule.^6,57^ We focus on the free-energy landscape defined by alanine dipeptide’s ϕ and ψ dihedral angles (see Figure 2a). To quantify the performance of BUQ, we employ two complementary metrics. First, we compare the free-energy surface calculated by our BUQ method after the *i*th query, *A*_*i*,BUQ_, to a thoroughly converged metadynamics reference, Â, which we treat as ground truth (see Supporting Information for details). We perform this comparison by computing the grid-based root-mean-squared deviation (RMSD) between *A*_*i*,BUQ_ and Â (see Supporting Information). A lower RMSD indicates a closer match to the benchmark, and a fast and consistent decrease of RMSD with the number of queries is desirable. Second, to compare the performance of BUQ to standard umbrella integration (UI), we perform UI on uniform grids of various resolutions, each with *l* = *m* × *m* umbrellas, yielding free-energy surfaces *A*_*l*,UI_. This allows for a fair comparison of MD computational cost, since constructing a BUQ surface *A*_*i*,BUQ_ requires the same number of MD steps as constructing a UI surface *A*_*l*,UI_ with *l* = *i*. We consider two UI variants: (i) standard UI without interpolation, where the free-energy gradient estimates are integrated directly, and (ii) a GP-interpolated UI, in which we postprocess the UI gradient estimates with a Gaussian process prior to integration, to obtain predictions of intermediate values, in the same spirit as ref 35 (see Figure 2b and Methods for details).

**Figure 2:**
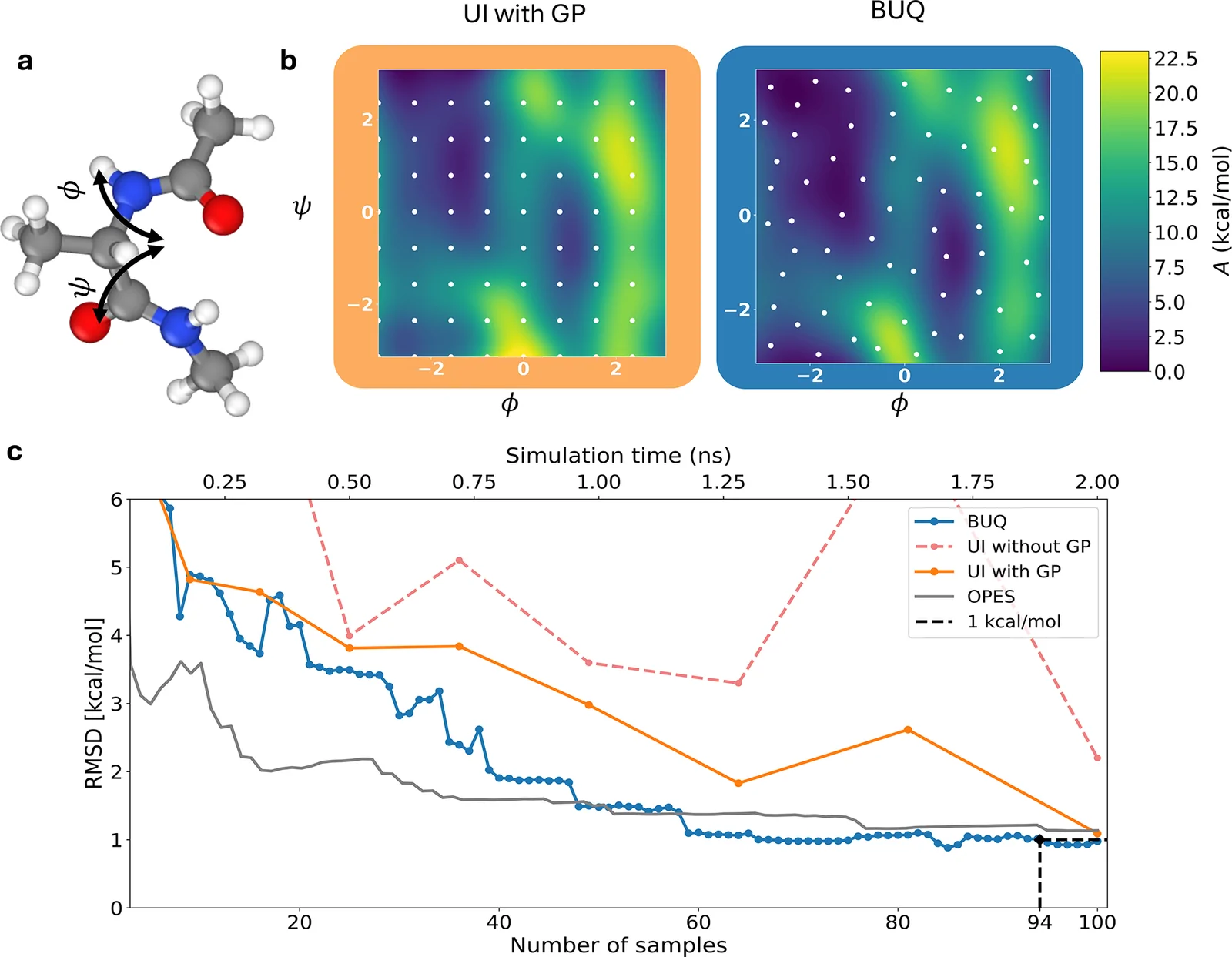
Bayesian Umbrella Quadrature (BUQ) sampling of the conformational free-energy landscape of alanine dipeptide. (a) Representation of the alanine dipeptide molecule with relevant collective variables (CVs), the dihedral angles ϕ and ψ. (b) Conformational free-energy landscape of alanine dipeptide in vacuo obtained by BUQ (blue) compared with an Umbrella Integration (UI) with Gaussian Process (GP) interpolation benchmark (orange). The distribution of umbrella centers (white markers) shows how BUQ adaptively places samples along the contours of valleys and barriers on the free-energy landscape. (c) Root-mean-squared deviation (RMSD) with respect to a ground-truth converged metadynamics reference as a function of the number of samples (bottom axis) and the corresponding simulation time (top axis). Shown are BUQ (blue), standard UI without GP-interpolation (dashed pink), UI with GP-interpolation (orange), and OPES (gray). The horizontal dashed black line marks 1 kcal/mol, and the vertical dashed black line indicates the BUQ query at which this threshold is first crossed. As measured by the RMSD with respect to the ground truth, BUQ consistently shows higher accuracy than UI with GP interpolation after 20 samples, and matches OPES accuracy after ∼1 ns of accumulated run time.

Finally, we compare BUQ to a modern adaptive biasing method, on-the-fly probability enhanced sampling (OPES). Via OPES, we calculate a free-energy surface using parameters inspired by ref 8, and we evaluate performance as a function of the same effective simulation time (up to 2 ns). We chose OPES as the main baseline for adaptive CV-based enhanced sampling because it provides a recent, well-tested implementation of on-the-fly bias construction designed to recover the underlying free-energy surface on the chosen CV space.^8^ OPES is readily available through PLUMED,^44,45^ has been applied to a broad range of molecular systems, and uses a small number of hyperparameters (e.g., the barrier parameter) that directly control the strength and evolution of the bias. This makes OPES a natural point of comparison for assessing the efficiency and robustness of BUQ under comparable conditions. We completed several OPES runs and kept the best-performing for comparison against BUQ (see Supporting Information for details).

In Figure 2c, we present BUQ sampling results compared against the benchmarks described above. BUQ was performed with a Matérn-5/2 kernel, a length scale of 0.75 rad, and a weight for exploiting free-energy minima λ = 0.1 (see Methods). For an overview of the hyperparameters, see Table 1. For a standard UI with a 10 × 10 umbrella grid, the free-energy surface RMSD relative to the metadynamics ground truth is ∼2.2 kcal/mol after 100 samples. BUQ reaches an RMSD of 2.2 kcal/mol after 39 samples, indicating a speedup of 2.6 with respect to standard UI. The GP-enhanced UI performs better, and reaches ∼1.1 kcal/mol after 100 queries, reflecting the benefit of GP interpolation, which BUQ matches in 83 queries, indicating a speedup of 1.2. Notably, BUQ surpasses the GP-aided UI ultimate performance and attains an RMSD of ∼1 kcal/mol after 94 queries, a level often considered chemical accuracy.^58^ For most of the run, and for all queries after query 20, BUQ’s RMSD is consistently lower than that of GP-interpolated UI at the same number of samples. This additional gain can be attributed to the IVR acquisition function. BUQ’s sampled points tend to follow the contours of valleys and barriers in the free-energy landscape, concentrating MD effort on the most informative regions.

**Table 1:** BUQ Hyperparameters for the Alanine Dipeptide Benchmark^a^

quantity	value
Collective variables	ϕ, ψ (dihedral angles)
Kernel	Matérn-5/2
Kernel length scale	0.75 rad (both CVs)
Umbrella force constant κ	200 kcal mol^–1^
Acquisition function	IVR, exploitation weight λ = 0.1
Simulation time per query	0.02 ns
Number of queries	100
Queries to reach 1 kcal/mol RMSD	94

a Here κ is applied to sin ϕ, cos ϕ, sin ψ, cos ψ, so it carries units of energy only, see Supporting Information.

When comparing BUQ to OPES under the same 2 ns simulation budget, we observe that OPES achieves rapid initial convergence and maintains a lower RMSD than BUQ for a substantial portion of the run (Figure 2c, gray curve). However, as sampling proceeds, BUQ catches up by the ∼1 ns mark, and, by the end of the 2 ns window, it attains a slightly lower RMSD than OPES and crosses the 1 kcal/mol threshold, whereas OPES remains close to but above this line. As we show in the Supporting Information, within this benchmark, OPES is also more sensitive to the choice of its hyperparameters (such as the barrier parameter) than BUQ is to the choice of its GP hyperparameters and restraining constant, κ.

Together, these results indicate that BUQ can accurately reconstruct the conformational free-energy landscape of a small biomolecule with faster and more robust convergence than standard UI and GP-interpolated UI, and with performance that is on par with a state-of-the-art biasing method such as OPES under equal MD computational budgets. Similar robust performance is obtained for variations of the BUQ kernel, hyperparameters, and acquisition functions, as reported in the Supporting Information.

### Phase Transition from Water to Ice

To demonstrate the generality of BUQ on a drastically different phenomenon, a nucleation process, we study the free-energy profile for the phase change from liquid water to ice Ih. The freezing of water has been a popular, relevant and challenging case study for enhanced sampling methods.^59–61^ Following Piaggi and Car,^43^ we use the TIP4P/Ice model and the environment similarity order parameter^62^ to distinguish between liquid- and ice-like water molecules. Put simply, the environment similarity is a measure of how closely the local neighbor arrangement of a specific atom matches a given template, e.g., that of ice Ih. In practice, we bias the number of water oxygen atoms with an environment similarity higher than a certain threshold with respect to ice Ih, which we label *N*_ice_. For more details see the Supporting Information.

In Figure 3, we show BUQ sampling results compared against variationally enhanced sampling (VES),^63^ as reported in ref 43. A full comparison of the RMSD with this benchmark is provided in the Supporting Information. BUQ was performed with a Matérn-1/2 kernel with a length scale of 20 *N*_ice_ and a weight for exploiting the free-energy minima λ = 0.1 (see Methods). For more hyperparameters, see Table 2. As initialization, we run four samples near the stable states (see Supporting Information for details). After 15 production samples, we find that the predicted free-energy profile closely resembles the one obtained via VES, with a difference in barrier height of 0.01 *NkT*, with *N* being the number of particles, *k* the Boltzmann constant, and *T* the temperature. The difference in the free-energy difference from the water to the ice state is 0.01 *NkT*. These residual discrepancies might reflect the different error mechanisms of dynamic and static biasing approaches: in a dynamical method such as VES, where walkers are free to sweep the CV space, an incomplete CV can lead to hysteresis and an accumulated biasing error.^7^ Contrastingly, in a static protocol such as BUQ, where samples are restrained to specific CV values, an overcomplete CV can overestimate free energies by effectively removing entropic contributions from degrees of freedom that are not essential to the transition.^64^ The latter might be the case with the nonrotationally invariant fixed template used as environment similarity CV, resulting in a slightly higher free-energy barrier and difference in BUQ than in VES. Additionally, the positions of the minima in the BUQ-based free-energy surface are slightly shifted relative to the VES reference, but they remain consistent with the stable-state locations identified independently from unbiased simulations (see Supporting Information). In other words, BUQ reproduces both the barrier and the stable states correctly. This shows that BUQ can sample a phase transition involving anisotropic molecules. In total, we run 19 × 15 ns = 285 ns. In comparison, ref 43 used 4 walkers, where each walker ran for 200 ns, giving 800 ns in total. This gives BUQ a remarkable 2.8 speedup considering the number of MD timesteps. Moreover, this calculation is promising for BUQ’s universality and ability to deal with landscapes with very rugged derivatives.

**Figure 3:**
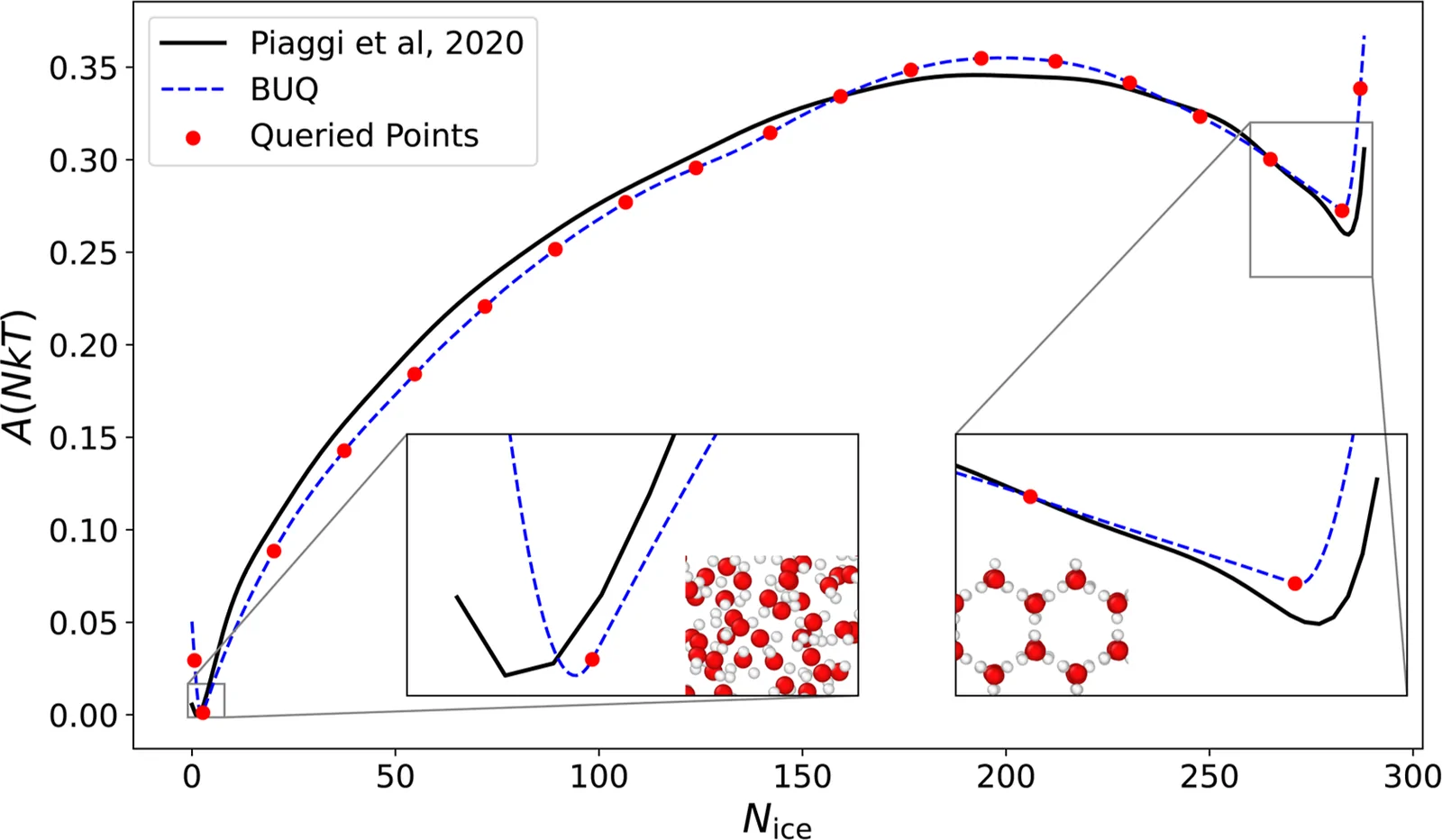
Bayesian Umbrella Quadrature (BUQ) sampling of the nucleation free-energy profile of the phase transition from water to ice Ih. Free energy as a function of the number of ice-like water molecules, *N*_ice_, obtained by BUQ (blue) compared against the reference profile from variationally enhanced sampling (black, based on data from the public repository associated with ref 43). BUQ samples are indicated by red dots.

**Table 2:** BUQ Hyperparameters for the Water-to-Ice Nucleation

quantity	value
Collective variable	*N*_ice_ (number of ice-like molecules)
Kernel	Matérn-1/2
Kernel length scale	20 *N*_ice_
Umbrella force constant κ	$100\;kJN_{ice}^{-2}$
Acquisition function	IVR, exploitation weight λ = 0.1
Simulation time per query	15 ns (total 19 × 15 ns = 285 ns)
Number of queries	19

### S_N_2 Chemical Reaction between Ethyl Chloride and a Fluoride Anion

Chemical-reaction free-energy landscapes are challenging; they are often rugged, high-dimensional, and reacting particles can move in an unbounded space.^65,66^ Moreover, even when using a machine-learning (ML) potential instead of density functional theory,^67^ the computation of the atomic interactions at each time step is orders of magnitude more costly than in classical MD. To further demonstrate the versatility of BUQ, we study the reaction free-energy landscape of an asymmetric S_N_2 substitution between ethyl chloride and a fluoride anion. We use a machine-learned reactive potential^68^ and describe the reaction with two CVs, *d*_1_ and *d*_2_, i.e., the distances from the carbon to the fluorine and chlorine atoms, respectively (see Figure 4). For each sampled point (*d*_1_, *d*_2_), we measure the forces and feed them to our BUQ workflow. We then apply the string method^69,70^ to the BUQ-predicted surface to extract the minimum free-energy path (MFEP) (for more details see the Supporting Information).

**Figure 4:**
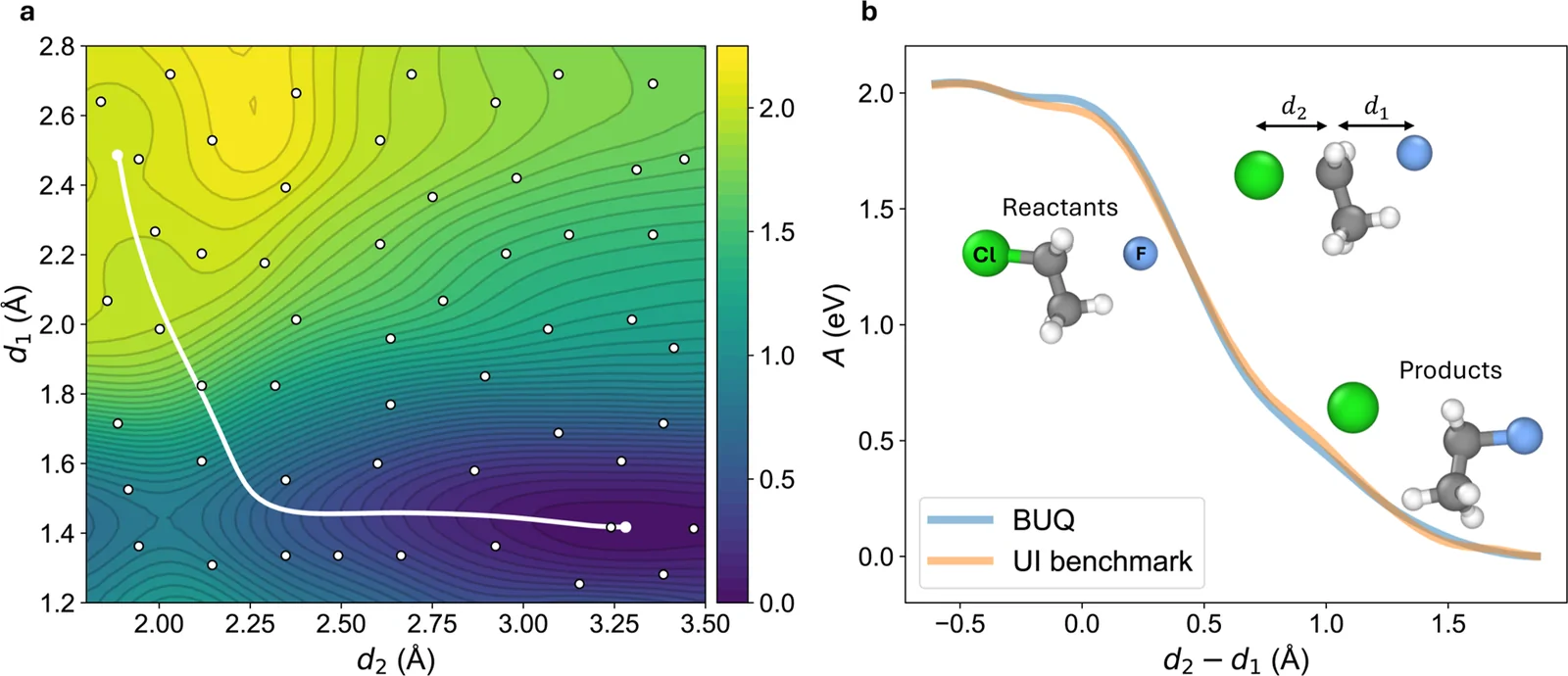
Bayesian Umbrella Quadrature (BUQ) sampling of the S_N_2 reaction free-energy landscape between ethyl chloride and a fluoride anion. (a) The free-energy surface is represented as a function of the collective variables *d*_1_ and *d*_2_, defined as the distances between the carbon atom and the fluorine and chlorine atoms in Å, respectively. Sampling points selected by BUQ are shown as white markers. The minimum free-energy path (MFEP), obtained via the string method, is shown in white. (b) Free energy along the MFEP according to BUQ (blue) and to the Umbrella Integration (UI) benchmark (orange), and the structures of different states of the reaction, indicating the reaction coordinates *d*_1_ and *d*_2_.

In Figure 4, we show BUQ sampling results, including the whole free-energy landscape and the reaction barrier. BUQ was performed with a Matérn-5/2 kernel and a length scale of 0.2 Å in both dimensions, and a weight for exploiting the free-energy minima λ = 0.2. For an overview of the hyperparameters, see Table 3. After 3 initial samples and 50 BUQ queries, the predicted free-energy barrier shows convergence with respect to the GP-interpolated UI benchmark consisting of 121 samples (see Supporting Information). Two BUQ umbrellas in which a hydrogen atom escaped into the gas phase were discarded and replaced by the closest available samples from the UI benchmark (see Supporting Information). The MFEP projected onto (*d*_1_, *d*_2_) agrees well with the minimum potential-energy path (MEP) reported in ref 68, with only small differences due to the widening of the free-energy minima at finite temperature. The reactants-to-products free-energy barrier of 0.005 eV is significantly lower than the reported potential-energy barrier of 0.261 eV. However, the BUQ free-energy barrier is consistent with the UI free-energy benchmark (0.008 eV) and spontaneous transitions from the reactant state seen in unbiased runs, see Supporting Information. This difference between the MFEP and the MEP barrier could be attributed to entropic contributions. However, other free-energy calculations in similar systems have reported barriers closer or greater than 0.3 eV,^71,72^ as well as potential-energy barriers closer to 0.2 eV,^73^ indicating that perhaps the ML potential requires additional refinement. Still, when considering this interaction potential, BUQ quickly converges and maps out the free-energy surface using few samples.

**Table 3:** BUQ Hyperparameters for the S_N_2 Reaction Benchmark

quantity	value
Collective variables	*d*_1_, *d*_2_ (C–F and C–Cl distances in Å)
Kernel	Matérn-5/2
Kernel length scale	0.2 Å in both *d*_1_ and *d*_2_
Umbrella force constants	$\kappa_{d_{1}}=1000\;{\mathrm{kcal}\;\mathrm{mol}}^{-1}{Å}^{-2}$
	$$\kappa_{d_{2}}=100\;\;{\mathrm{kcal}\;\mathrm{mol}}^{-1}{Å}^{-2}$$
Acquisition function	IVR, exploitation weight λ = 0.2
Simulation time per query	40 ps
Number of queries	53

## Discussion

We have developed Bayesian Umbrella Quadrature (BUQ), a more efficient and universal free-energy calculation method combining the well-known Umbrella Integration (UI) framework with Bayesian quadrature (BQ). BUQ overcomes two of the largest challenges in standard UI: (1) interpolating the free energy between gradient samples and (2) selecting where to place these samples. In our approach, the free-energy gradient is modeled by a Gaussian process (GP)—i.e., a noise-tolerant probabilistic model—while an acquisition function iteratively proposes the most informative gradient samples to reduce the variance of the integral, i.e., the free energy. In other words, instead of uniformly covering the CV-space with numerous windows, we iteratively choose the next bias location that maximally improves our estimate of the free energy. To our knowledge, this is the very first application of BQ to enhanced sampling MD simulations. Our tests show that this adaptive BUQ strategy converges significantly faster than standard, i.e., uniform, UI. This is first shown in the (ϕ, ψ) conformational free-energy calculation of alanine dipeptide, where BUQ has a 2.6 sample-efficiency speedup compared to standard UI and a 1.2 speedup with respect to UI with a GP interpolation (Figure 2). This speedup can be explained by the two aforementioned characteristics of BUQ. On one hand, the GP provides a smooth model of the free-energy gradient that can interpolate between samples. Prior studies have shown that GP-based reconstruction can greatly reduce cost by exploiting correlations in the free-energy data.^33–35^ On the other hand, the acquisition function effectively samples key regions—for example, near barriers or changes in curvature. Our results confirm that a Bayesian sampling strategy can improve MD-based free-energy calculations, in agreement with the broader literature on probabilistic numerics.^56,74^ We also benchmark BUQ against the modern adaptive biasing method OPES, finding that, under the same 2 ns simulation budget, BUQ remains competitive and attains a slightly lower final RMSD with respect to the reference, while OPES exhibits faster initial convergence. When comparing how sensitive each method is to its settings, OPES shows a stronger sensitivity to its barrier parameter than BUQ to its GP hyperparameters or its force constant (see Figure 2 and Supporting Information).

To demonstrate that BUQ has transferable performance across systems and processes, we apply the method to two additional, fundamentally different, rare events. First, we tackle the phase-change from water to ice Ih, described by the number of ice-like water molecules, *N*_ice_, as determined by an environment similarity order parameter. BUQ performance shines again. Compared to variationally enhanced sampling, it delivers a 2.8 speedup considering the total number of MD timesteps. The nucleation free-energy barriers agree within 0.1 *NkT*, a small discrepancy attributable to the difference between static and dynamic biasing methods. Second, we study an asymmetric *S*_N_2 chemical reaction between ethyl chloride and a fluoride anion. Even when using an ML potential, computing each new time step can be far more expensive than when using classical force fields. Here, BUQ invests only 53 samples to compute a reaction free-energy landscape, which validates well against a UI benchmark and related literature. Our results show how BUQ can become a general tool across different domains and phenomena, including structural transitions, phase equilibria, and reaction pathways.

One practical challenge when deploying BUQ is choosing its hyperparameters, such as the type of kernel or length scale for the GP.^55^ First, we note that similar requirements arise in all enhanced-sampling methods, such as the placement of the umbrellas and the force constants in US or UI;^9,14^ the choice of the Gaussian width, height, and deposition frequency in metadynamics;^6,7^ or the barrier parameter in OPES.^8^ In BUQ, the most relevant hyperparameters are those that govern the acquisition strategy and the surrogate model, i.e., the choice of acquisition function, the exploitation weight λ, and the GP kernel type and length scale, which together dictate where new samples are placed. For alanine dipeptide, both IVR and uncertainty sampling produced similarly good convergence, with IVR being slightly superior (see Supporting Information). For all other systems, we therefore employed IVR exclusively. The exploitation weight should not be set too high, otherwise sampling collapses into the minima. In practice, λ = 0.1 or 0.2 provided a good balance, with λ = 0.1 performing slightly better for alanine dipeptide and the phase transition, whereas λ = 0.2 performed slightly better for the S_N_2 system, which has steeper free-energy valleys. Regarding the GP kernel, we found Matérn kernels, and in particular Matérn-5/2, to be a robust default choice across the different benchmarks. An RBF kernel has comparable results (see alanine dipeptide tests in the Supporting Information). For the water–ice transition, where the free-energy derivative is highly rugged, a Matérn-1/2 kernel was required to capture the sharper features. The GP length scale is also important: if chosen too large, the model enforces unrealistically long-range trends; if too small, it overfits local noise. A practical rule of thumb in our examples is to set the length scale to roughly 1 order of magnitude smaller than the CV range. Moderate changes around this value do not significantly affect the results (as illustrated for alanine dipeptide in the Supporting Information). The MD simulation length per query is another hyperparameter: longer trajectories provide better statistics and more reliable estimates of the mean force, at the cost of additional compute. The length of each MD run and the convergence of the estimated gradient can be assessed via block analysis.^75^ Regarding BUQ’s initialization, we typically place BUQ windows at free-energy minima identified during equilibrations runs, but for the water–ice case it was important to also include extra windows adjacent to the stable states to better capture the steep changes in the derivative (see Supporting Information). When initial samples are close to the boundaries of the CV range, BUQ’s acquisition will avoid resampling these regions as they have little effect on the IVR, so it is important to locate these initial samples judiciously. Finally, we observe that adding more BUQ queries leads to clear gains at early stages, but with diminishing returns once the main features of the landscape are resolved, as seen in the RMSD convergence curves (Figure 2 and Supporting Information).

One advantage of BUQ is that the collected data points can always be reprocessed with a different GP setup without rerunning biased MD simulations, enabling a posteriori changes in kernel type or length scale and selective extension of trajectories at critical CV values if needed. Furthermore, the restraining constant, κ, which is crucial in conventional US because it controls both overlap and stability, has a reduced influence in UI-based BUQ: the reconstructed free-energy surfaces and convergence behavior are only weakly affected by its value (see Supporting Information). Overall, we find that many different BUQ hyperparameter choices lead to successful free-energy calculations, indicating that the method is reasonably robust. In this work, BUQ hyperparameters were tuned manually; a more systematic exploration and automated selection strategy is an important direction for future work. Another limitation of all enhanced sampling methods is diagnosing convergence; because BUQ provides a posterior distribution on the free energy, we have a measure of uncertainty, but turning that into a clear stopping criterion is nontrivial. In practice, one must still inspect the evolving free-energy surface for convergence. Same as in standard UI, one can use error analysis to monitor the unimodality and consistency of the biasing force. Also like in standard UI, steering the system to a target window can occasionally fail if the bias is too weak to overcome a barrier, or if the steering occurs too quickly. Finally, BUQ, same as UI or US, assumes that the CVs are well-chosen; as always, if important motions lie outside the chosen variables, the free energy will not be captured accurately.

Looking forward, there are many BUQ extensions to explore in future work. In this study, we focused on getting a reliable free-energy landscape overall. However, with small adaptations to the acquisition function, BUQ can also be used to focus on stable basins or transition states. BUQ’s acquisition function could also be used to, for example, guide the refinement of an ML potential at regions when one wishes to improve a free-energy estimate. Moreover, multifidelity Bayesian strategies^76^ offer an avenue to compute free energies at varying accuracies and computational costs. When looking at the GP, one can explore many more types of kernels, including quantum ones,^77,78^ whose expressivity might be better suited for certain free-energy landscapes. BUQ’s probabilistic framework can also be extended to multiple related integrals,^79^ which could be useful when studying similar molecules or materials. For example, one could simultaneously compute free-energy surfaces for the same system at different temperature or pressure conditions, accelerating high-throughput screening. Moreover, a nonharmonic functional form of the bias could also be evaluated, such as Gaussians or polynomials, enabling exploration with a single simulation and possibly leading to even faster convergence of the free energy. In this work, we restrict ourselves to one- and two-dimensional CV spaces, which are fairly common in practice and whose efficient calculation is still very useful for the molecular simulation community. We emphasize that the challenge of selecting appropriate CVs for complex, high-dimensional systems, such as mini-proteins or other large biomolecular assemblies, is a general one, shared by all biased sampling methods and not specifically addressed by BUQ in its current form. Extending BUQ to higher-dimensional integrals in complex CV spaces remains challenging but highly rewarding. Developing scalable BUQ strategies is therefore an important direction for future work. Interfacing BUQ with deep-learned CVs^80^ or path CVs^81^ are possible avenues to mitigate dimensionality, as well as the adaptation of strategies for high-dimensional Bayesian optimization.^82^ Finally, beyond the three types of rare events showcased here, our open-source BUQ framework eases the application to further transitions, such as alchemical or binding free-energy calculations with appropriate CVs. In summary, by demonstrating fast convergence and broad applicability, our BUQ pipeline opens the door to a new generation of automated free-energy methods that combine enhanced sampling and Bayesian inference.

## Supplementary Material



## Data Availability

Code to reproduce all analysis and results is available at: https://github.com/computational-chemistry-uva/BUQ.

## Notes

Details on Bayesian Quadrature (Gaussian processes, acquisition functions), molecular dynamics and biasing protocols (PDF)
