# Supplementary material for: Bayesian Umbrella Quadrature Automates Free-Energy Calculations Across Diverse Molecular Systems and Processes
Source: J Chem Theory Comput. 2026 Jun 11;22(18):9332–42. doi: 10.1021/acs.jctc.6c00120 (PMC13602914; doi:10.1021/acs.jctc.6c00120)
Supplement: Supplementary file 1 [file ct6c00120_si_001.pdf]

Supporting Information: Bayesian umbrella quadrature automates free-energy calculations across diverse molecular systems and processes

Eline K. Kempkes and Alberto Pérez de Alba Ortíz*

Computational Soft Matter Lab, Computational Chemistry Group and Computational Science Lab, Van 't Hoff Institute for Molecular Sciences and Informatics Institute, University of Amsterdam, Science Park 904, 1098 XH Amsterdam, The Netherlands.

E-mail: a.perezdealbaortiz@uva.nl

S.1 Gaussian Process Surrogate Models

The choice of covariance kernel determines the prior smoothness assumptions about the mean biasing force, i.e., the free-energy gradient. In this study, we have mainly used three different kernels: the Radial Basis Function (RBF), Matérn-1/2 and Matérn-5/2.[1] We employ stationary kernels with automatic relevance determination (ARD), allowing each collective variable (CV) dimension to have its own characteristic lengthscale. On top of these three kernels, it is possible to add a white-noise term to represent measurement noise originating from finite sampling in the biased Molecular Dynamics (MD) simulations. In this section, we will shortly discuss the different kernels.

S.1.1 Radial Basis Function (RBF) Kernel

The RBF, also known as squared–exponential kernel assumes an infinitely differentiable underlying function. For CVs $\mathbf{s}, \mathbf{s}' \in \mathbb{R}^d$,

$$k_{\text{RBF}}(\mathbf{s}, \mathbf{s}') = \sigma^2 \exp\left(-\frac{1}{2}\sum_{j=1}^{d}\frac{(s_j - s'_j)^2}{\ell_j^2}\right). \tag{1}$$

Here, the hyperparameters are ℓ_j, the lengthscale in dimension j, and σ^2, the process variance. The RBF kernel imposes strong smoothness, the integrand and all derivatives exist and are continuous. When added to a white-noise component

$$k_{\text{white}}(\mathbf{s}, \mathbf{s}') = \tau^2 \delta_{\mathbf{ss}'}, \tag{2}$$

the full kernel becomes $k = k_{\text{RBF}} + k_{\text{white}}$. When setting the noise-parameter $\tau = 0$, it reduces to just an RBF kernel.

S.1.2 Matérn Kernels

The Matérn family provides controlled smoothness via the parameter ν. The Matérn kernel reduces to RBF in the limit $\nu \to \infty$. Using ARD lengthscales with Euclidean metric, we define

$$r(\mathbf{s}, \mathbf{s}') = \sqrt{\sum_{j=1}^{d}\frac{(s_j - s'_j)^2}{\ell_j^2}}. \tag{3}$$

The full Matérn kernel with smoothness parameter ν is given by

$$k_{\text{M}\nu}(\mathbf{s}, \mathbf{s}') = \sigma^2 \frac{2^{1-\nu}}{\Gamma(\nu)}\left(\sqrt{2\nu}\, r\right)^{\nu} K_{\nu}\left(\sqrt{2\nu}\, r\right), \tag{4}$$

where r is defined in Eq. 3, σ is the variance, $\Gamma(\cdot)$ denotes the Gamma function, and $K_\nu(\cdot)$ is the modified Bessel function of the second kind. The Matérn-1/2 kernel describes non-

differentiable functions and is equivalent to the exponential kernel:

$$k_{\mathrm{M12}}(\mathbf{s}, \mathbf{s}') = \sigma^2 \exp(-r), \tag{5}$$

with r defined as in Eq. 3 and σ is again the variance. This kernel sets $\nu = 1/2$ and imposes minimal smoothness, which allows for sharp variation in the mean force profile. In contrast, the Matérn-5/2 kernel (i.e. with $\nu = 5/2$) corresponds to twice differentiable sample paths. It is defined as:

$$k_{\mathrm{M52}}(\mathbf{s}, \mathbf{s}') = \sigma^2 \left(1 + \sqrt{5}r + \frac{5}{3}r^2\right) \exp(-\sqrt{5}r), \tag{6}$$

with r defined as in Eq. 3 and σ is the variance. The Matérn-5/2 kernel is suitable when the mean force is smoother than once-differentiable fields but not as smooth as RBF. Equivalent to the RBF, we can also add the white kernel to represent the noise from the MD sampling, so the full model uses $k = k_{\mathrm{M}} + k_{\mathrm{white}}$.

S.1.3 Practical Implementation

In all cases, the GP is constructed with ARD kernels over a maximum of two CV dimensions, where a white-noise term can be added for measurement uncertainty. Kernel selection controls the prior regularity of the free-energy gradient (i.e., mean biasing force). RBF favors globally smooth surfaces, while Matérn variants allow increasingly rough behavior. All kernels are defined using the GPY library[2] and then wrapped in the Emukit[3,4] library. For all Emukit kernels, we use the Lebesgue measure, which is a uniform distribution.

S.2 Acquisition Functions

Acquisition functions guide the sequential selection of new sampling locations in Bayesian Quadrature (BQ) .[5,6] At each iteration, the next CV point is chosen by maximizing the

acquisition that quantifies the expected utility of evaluating the mean force at that location. All strategies considered here depend directly on the GP posterior. We describe two classes: uncertainty sampling and integral–variance–reduction (IVR), as well as the free-energy minima exploiting term used in the main text.

S.2.1 Uncertainty Sampling

Uncertainty sampling selects points where the GP predictive variance of the integrand is maximal .[6] For a GP surrogate of $-\mathbf{F}(\mathbf{s})$ with posterior variance $k_D(\mathbf{s}, \mathbf{s})$, the acquisition is simply

$$a_{\mathrm{US}}(\mathbf{s}) = k(\mathbf{s}, \mathbf{s}) \cdot (p(\mathbf{s}))^q. \tag{7}$$

Here, $p(\mathbf{s})$ is the density of the integration measure, and q is the measure power parameter. The default choice in Emukit is $q = 2$. When the integration measure is uniform (Lebesgue), it reduces to:

$$a_{\mathrm{US}}(\mathbf{s}) \propto k(\mathbf{s}, \mathbf{s}), \tag{8}$$

reducing to the classical predictive–variance criterion widely used in uncertainty sampling.

This prioritizes locations where the surrogate of the mean force is least certain, independent of their impact on the integral. Uncertainty sampling is computationally inexpensive and provides broad exploration when little is known about the landscape. In quadrature problems, it can oversample regions irrelevant for the integral, motivating criteria that consider both predictive variance and relevance to the integral estimate. However, since it is economical and easily implemented, we tested it as well.

S.2.2 Integral Variance Reduction (IVR)

The IVR strategy selects points expected to maximally reduce the posterior variance of the integral.[5] The acquisition is the expected drop in integral uncertainty when augmenting the

dataset with a new observation:

$$a_{\mathrm{IVR}}(\mathbf{s}) = \mathbb{V}n - \mathbb{V}\big[A \mid D \cup \mathbf{s}\big]. \tag{9}$$

Here, $\mathbb{V}n$ is the current variance of A, and $\mathbb{V}\big[A \mid D \cup \mathbf{s}\big]$ is the variance when the next observation $\mathbf{s}$ is sampled. This may also be written as the squared correlation $\rho^2(\mathbf{s})$ between the integrand value at $\mathbf{s}$ and the integral. IVR thus focuses sampling in regions that most influence the quantity of interest rather than simply uncertain locations.

S.2.3 Exploitation of free-energy minima

To accelerate the finding of minimum free–energy pathways, we combine IVR or uncertainty sampling with a term that directs sampling toward predicted low–free–energy regions. If $a_{\mathrm{FES}}(\mathbf{s})$ denotes the normalized GP estimate of $A(\mathbf{s})$ and $\lambda \in [0, 1]$ controls trade–off,

$$a_{\mathrm{total}}(\mathbf{s}) = -\lambda a_{\mathrm{FES}}(\mathbf{s}) + (1 - \lambda) a_{\mathrm{IVR|US}}(\mathbf{s}). \tag{10}$$

Here, both $a_{\mathrm{IVR|US}}$ and the current FES estimate a_{FES} are normalized. The first term favors exploitation of likely low–free–energy states, while the second enforces global exploration via variance reduction of the integral (IVR) or variance reduction of the force profile (Uncertainty Sampling). For $\lambda = 0$, the strategy reduces to pure IVR or pure uncertainty sampling, whereas $\lambda \to 1$ favors pure exploitation. This combined approach suppresses unnecessary sampling in high–free–energy regions and accelerates refinement in physically relevant regions.

S.3 Conformational change in alanine dipeptide

S.3.1 Molecular Dynamics protocol

To establish a benchmark, we carried out simulations of alanine dipeptide in vacuum. An initial unbiased molecular dynamics trajectory of 5 ns was done with GROMACS 2022.5,[7] to equilibrate the system. The system was simulated at 300 K using the AMBER99SB-ILDN[8] force field and the Verlet cutoff scheme with PME electrostatics (real-space tolerance 1×10^{-5}; Lennard-Jones tolerance 0.001). The Coulomb cutoff and the van der Waals cutoff were both set to 1 nm. The cubic box had an edge length of 2.72 nm. Bond constraints on all hydrogen-involving interactions were imposed using LINCS.[9] The integration timestep was 2 fs, and temperature was controlled with the velocity rescale thermostat with a time constant of 0.1 ps.[10] For the rest of the discussion of alanine dipeptide, the same settings are used, unless specified otherwise. The result of this 5 ns unbiased MD was used as a starting point for the metadynamics and for the umbrella sampling runs.

S.3.2 Metadynamics protocol

To get a benchmark, we performed a 100 ns metadynamics simulation in Gromacs 2023 using PLUMED 2.9.1.[11,12] As CVs, we used the backbone dihedral angles ϕ and ψ, with Gaussian potentials deposited every 2 ps. The Gaussian height was 0.1 kcal/mol with a width of 0.25 rad in each CV. All PLUMED input files are provided in the repository.

To assess the convergence of the benchmark metadynamics simulation, we analyzed the generated free energy surfaces (FES), after the deposition of each Gaussian. To assess convergence, a block-averaging scheme was employed, using blocks of 500 and 1000 FES files (1 ns and 2 ns, respectively). For each block, the free energies were averaged and aligned to zero at their minimum. Convergence was quantified via the root-mean-square deviation (RMSD) relative to the final block, see Fig. S1. For the last 50 ns, the largest deviation of the FES from the final 2 ns (the final FES-block average), is 0.52 kcal/mol. This block-average

of the final 2 ns is taken as the benchmark of the Bayesian quadrature results.

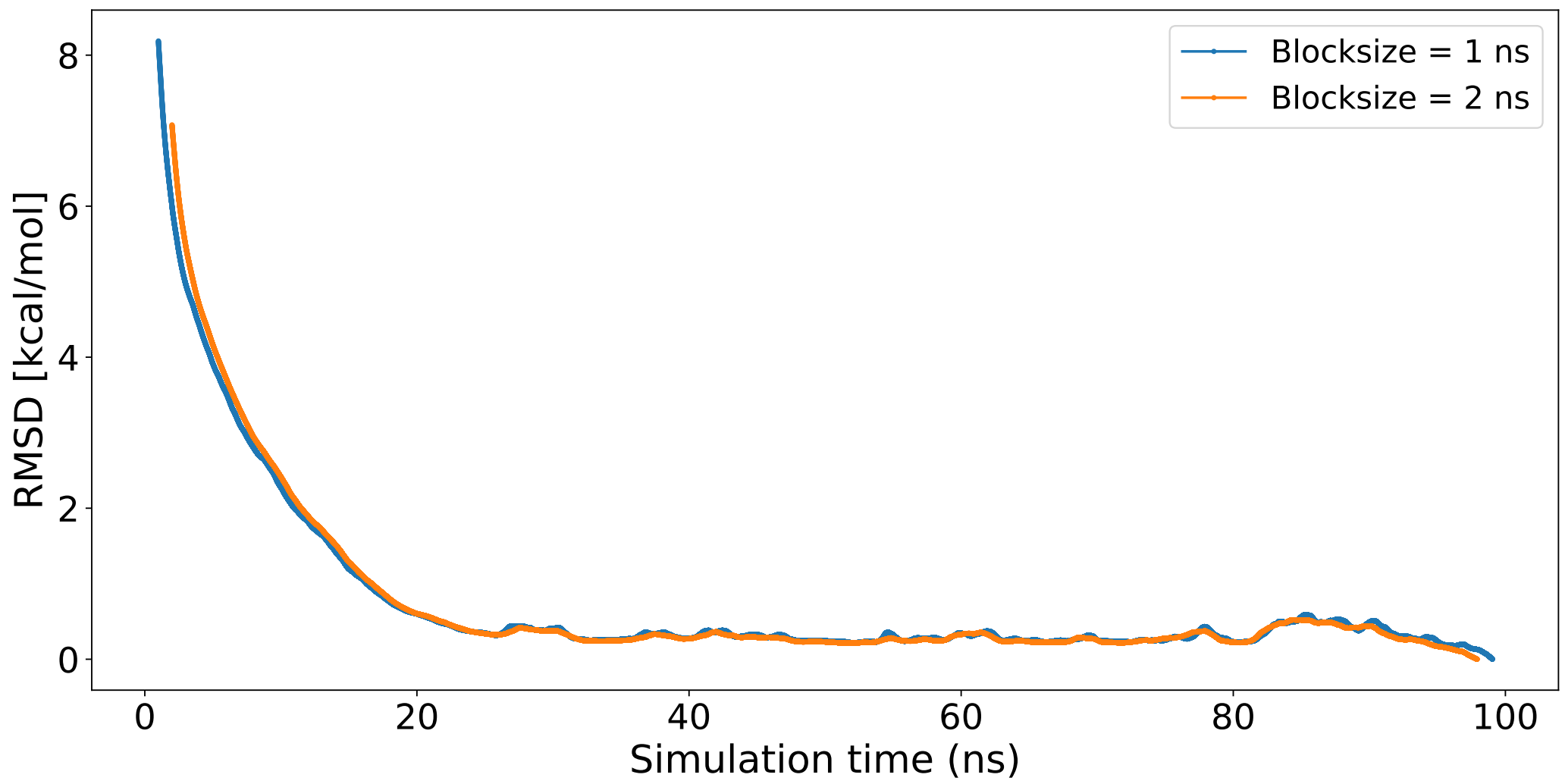

Figure S1: Block-averaging convergence analysis of the benchmark metadynamics free energy surface (FES). FES estimations were averaged over blocks of 500 (1 ns) and 1000 (2 ns) Gaussian depositions, aligned to zero at their minimum, and compared to the final block via RMSD.

S.3.3 Umbrella Sampling protocol

For the US and BUQ samples, we run short restrained MD at different target values of $(\phi^\star, \psi^\star)$. These biased simulations are done with GROMACS 2023[7] patched with PLUMED 2.9.1.[11] The simulation time was 0.02 ns per sample, or 0.2 ns per sample for the longer simulations. All MD simulation settings are as described in S.3.1, and all samples are started from the equilibrated state. PLUMED input files declared `UNITS LENGTH=A TIME=ps ENERGY=kcal/mol` and defined the backbone torsions ϕ and ψ together with their trigonometric components $\{\sin\phi, \cos\phi, \sin\psi, \cos\psi\}$, which ease the biasing or periodic CVs. `MOVINGRESTRAINT` harmonic biases were applied on the four trigonometric CVs with the same force constant $\kappa = 200$ kcal mol^{-1} rad^{-2}. The biasing schedule is as follows:

1. an unbiased start (500 timesteps) (only for the longer simulations of 0.2 ns)

2. gradual increase of the force constant up to the target κ by step 1000 (for the simulations of 0.02 ns) or 1500 (for the simulations of 0.2 ns)

3. smooth translation of the bias centers from the initial values $(\phi_0, \psi_0) = (-1.580543,\ 1.311767)$ to the target window $(\phi^\star, \psi^\star)$ at a speed proportional to the change in angles, $|\Delta\theta|$, completed by step $1000 + 1000\,|\Delta\theta|$ or $1500 + 1000\,|\Delta\theta|$ in case of the longer runs.

Each query consisted of $N_{\text{steps}} = 10{,}000$ MD timesteps (20 ps total), or $N_{\text{steps}} = 100{,}000$ MD timesteps (200 ps total) and we used the last 10 or 100 ps for measurement of the biasing force. PLUMED printed $\sin\phi$, $\cos\phi$, $\sin\psi$, $\cos\psi$ every 100 steps to a file. Convergence of the force was verified via block analysis.

From the time-averaged trigonometric CVs $(\overline{\sin\theta}, \overline{\cos\theta})$ and the restraint targets $(\sin\theta^\star, \cos\theta^\star)$ for $\theta \in \{\phi, \psi\}$, we formed the two harmonic forces $f_{\sin\theta} = k_\theta(\overline{\sin\theta} - \sin\theta^\star)$ and $f_{\cos\theta} = k_\theta(\overline{\cos\theta} - \cos\theta^\star)$ and projected them onto the angular direction using the phase difference via atan2:

$$F_\theta = -\ \mathrm{sgn}\Big(\ \mathrm{atan2}(\overline{\sin\theta}, \overline{\cos\theta}) - \mathrm{atan2}(\sin\theta^\star, \cos\theta^\star)\Big)\ \sqrt{f_{\sin\theta}^2 + f_{\cos\theta}^2},$$

yielding the desired noisy estimate of $(-\partial_\theta A)$ at $(\phi^\star, \psi^\star)$.

S.3.4 Obtaining and benchmarking a free-energy landscape from BUQ

In these simulations, angles were treated as periodic with domain $\phi, \psi \in [-\pi, \pi)$, and all energies are in kcal/mol, times in ps, angles in radians. To obtain a free-energy surface from BUQ, we modeled the 2D gradient field with a Gaussian process using **GPy**[2] and **Emukit**.[3,4] A small diagonal white kernel component could be added to model measurement noise. The GP was wrapped in Emukit's quadrature interface with a Lebesgue prior over $[-\pi, \pi]^2$ and trained on the acquired pairs $\{(\phi_i, \psi_i), (-F_{\phi,i}, -F_{\psi,i})\}$. We then predicted the gradient on

a 100×100 grid and integrated it to a free energy using a composite trapezoid rule on the grid, followed by an L-BFGS-B refinement that minimizes the squared mismatch between the numerical gradient of the surface and the GP-predicted gradient.[13] The resulting free-energy surface was shifted to have minimum zero. Accuracy was quantified by the root mean squared Deviation (RMSD) with respect to the ground-truth metadynamics reference, projected onto the same grid points:

$$\mathrm{RMSD} \;=\; \sqrt{\frac{1}{10000}\sum_{i=1}^{100}\sum_{j=1}^{100}\Big(A_{\text{prediction}}(s_{ij}) - A_{\text{metadynamics}}(s_{ij})\Big)^2}. \tag{11}$$

The same procedure is done to obtain and benchmark free-energy surfaces from the standard UI simulations with uniform grids. In these cases, the pairs $\{(\phi_i, \psi_i), (F_{\phi,i}, F_{\psi,i})\}$ are determined by the uniform grid.

Here we provide details about both cases:

1. BUQ: We initialized with two samples at the minima of the surface, $(\phi, \psi) = (-1.508,\ 0.880)$ and $(1.194,\ -0.880)$ and updated the GP after each new measurement. We evaluated two acquisitions: Integral Variance Reduction (IVR) and Uncertainty Sampling. We have used this with both RBF and Matérn-5/2 kernels, varying the lengthscale and the minima exploitation parameter λ (see below). We repeated this sampling for a fixed budget of 98 queries, i.e., 100 samples in total.

2. Standard UI on uniform grid: We queried an $n \times n$ lattice with $n = 1, \ldots, 10$ (i.e., from 1 to 100 grid points). We used this data to obtain the UI without GP benchmark, where we did not use any interpolation of the grid, e.g., the 5x5 grid had just 25 datapoints to integrate. Then, we used a regular grid interpolation to predict it on a 100x100 grid, to compare with the metadynamics surface. For the UI with GP, we used an RBF kernel with lengthscale $\ell = 0.5$ and no additional white noise. For fairness, each query used the same MD budget described above. RMSD was reported as a function of n^2 queries. To consider periodicity, points sampled exactly at a dihedral angle of

$-\pi$ were duplicated on the opposite boundary.

S.3.5 Bayesian Umbrella Quadrature protocol

To assess hyperparameters, we performed several BUQ runs, see Fig. S2, where we varied the lengthscale $\ell \in \{0.5, 0.6\}$ rad, for the RBF kernel, and we set $\ell = 0.75$ rad for the Matérn-5/2 kernel. We also used two different acquisition functions, uncertainty sampling and integral variance reduction (IVR). In this section, all the simulations last for 0.2 ns. We also implement white-noise variance $\in \{0, 0.1\}$ (only for the Matérn-5/2) and acquisition $\in \{\mathrm{IVR}, \mathrm{US}\}$, (for both kernel types) and $\lambda \in \{0, 0.1, 0.2\}$ (for both kernel types), all with 100 queries. These settings were decided after an initial exploration. The best-performing configuration (lowest RMSD after 100 queries) was: Matérn-5/2 kernel (ARD) with length-scales $\ell = 0.75$, zero added white noise, IVR acquisition with $\lambda = 0.1$, and 100 sequential queries. This setting was used for the BUQ results in the main text.

S.3.6 Comparing BUQ to OPES

To compare BUQ with on-the-fly probability enhanced sampling (OPES), we ran a series of OPES simulations using input parameters based on Ref.[14] and example files provided with the method. In the original setup, the OPES barrier parameter is set to 50 kJ/mol; here, we additionally considered half and twice this value, i.e., barrier parameters of 25, 50, and 100 kJ/mol, all with a bias update frequency of `PACE` = 500. Each OPES simulation was run for 100 ns in total. For a fair comparison with BUQ, we restricted the analysis to the first 2 ns, corresponding to the same effective simulation time used by BUQ. With respect to BUQ, we use umbrellas with short simulation time (0.02 ns), Matérn-5/2 kernel $\ell = 0.75$ rad and $\lambda = 0.1$.

For each OPES setup, we reconstructed the FES in the (ϕ, ψ) dihedral space and monitored its convergence by computing the root-mean-square deviation (RMSD) with respect to the converged metadynamics benchmark FES used as reference. The RMSD was evaluated

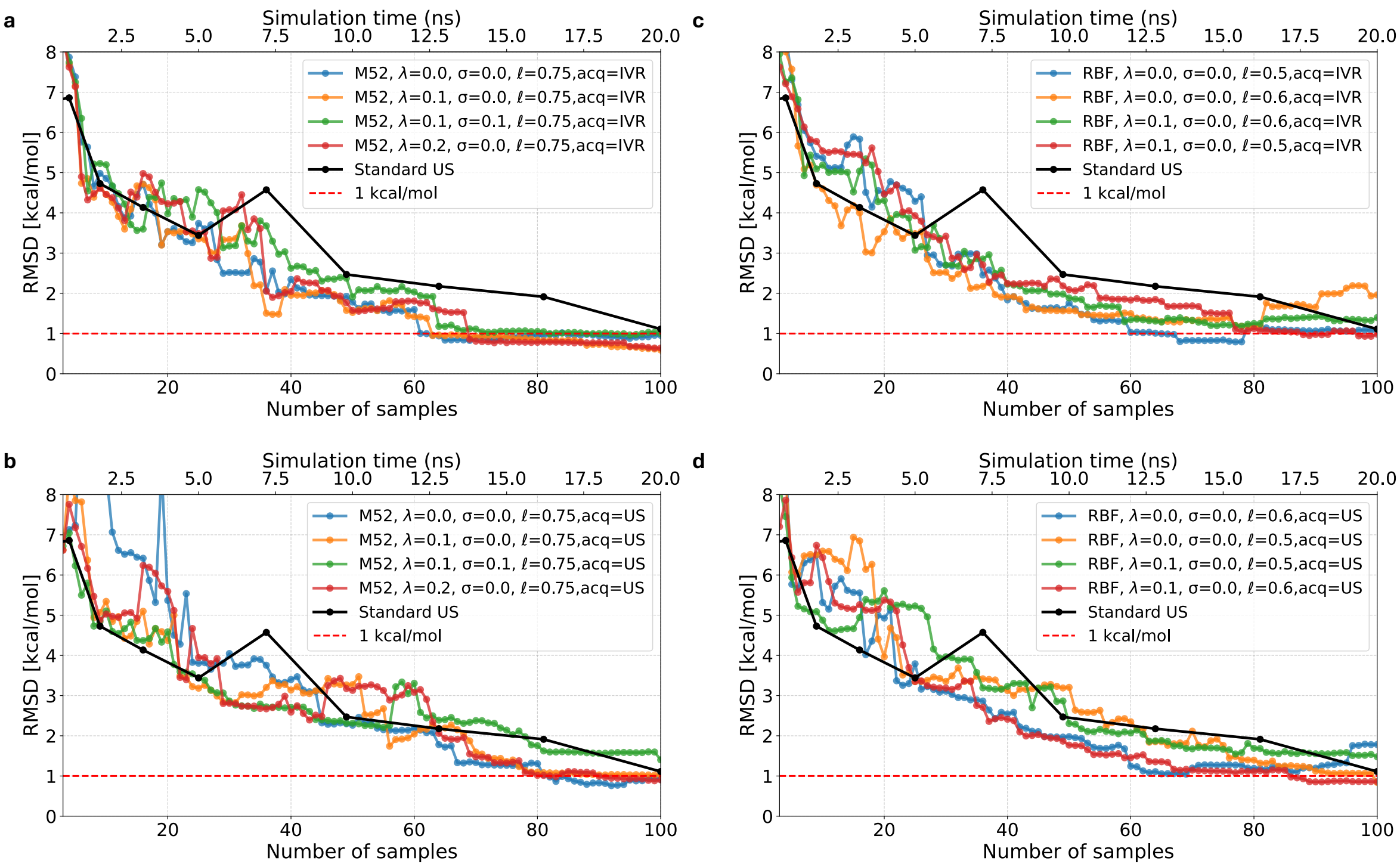

Figure S2: Free-energy landscape root-mean-square deviation (RMSD) with respect to the metadynamics ground truth as a function of the number of Bayesian umbrella quadrature (BUQ) queries with different hyperparameters. (a) Matérn-5/2 kernel with integral variance reduction (IVR) acquisition. (b) Matérn-5/2 kernel with uncertainty sampling (US in the legend). (c) RBF kernel with IVR acquisition. (d) RBF kernel with uncertainty sampling, where the lengthscale was varied as $\ell \in \{0.5, 0.6\}$ rad, while for the Matérn-5/2 kernel $\ell = 0.75$ rad was used. The white-noise variance was $\sigma \in \{0, 0.1\}$ for Matérn-5/2 only. The exploitation parameter was $\lambda \in \{0, 0.1, 0.2\}$ for both kernel types. Each configuration was evaluated with 2 initial samples followed by 98 acquired samples. Black curves denote standard umbrella sampling (US) on a uniform grid, and the red dashed line indicates the 1 kcal/mol target accuracy.

after different simulation times, and the same RMSD metric was computed for BUQ as a function of the number of BUQ samples.

The OPES run with barrier 100 kJ/mol shows the fastest decrease in RMSD among the three OPES variants; almost reaching the 1 kcal/mol threshold. The barrier of 50 kJ/mol, which was used in Ref.,[14] converges more slowly and plateaus around an RMSD of $\sim$ 2 kcal/mol, while the smallest barrier of 25 kJ/mol remains at significantly larger RMSD values. In contrast, BUQ with $\kappa = 200$ reaches sub–1 kcal/mol RMSD within the same effective simulation budget, indicating more efficient convergence of the FES in this test case.

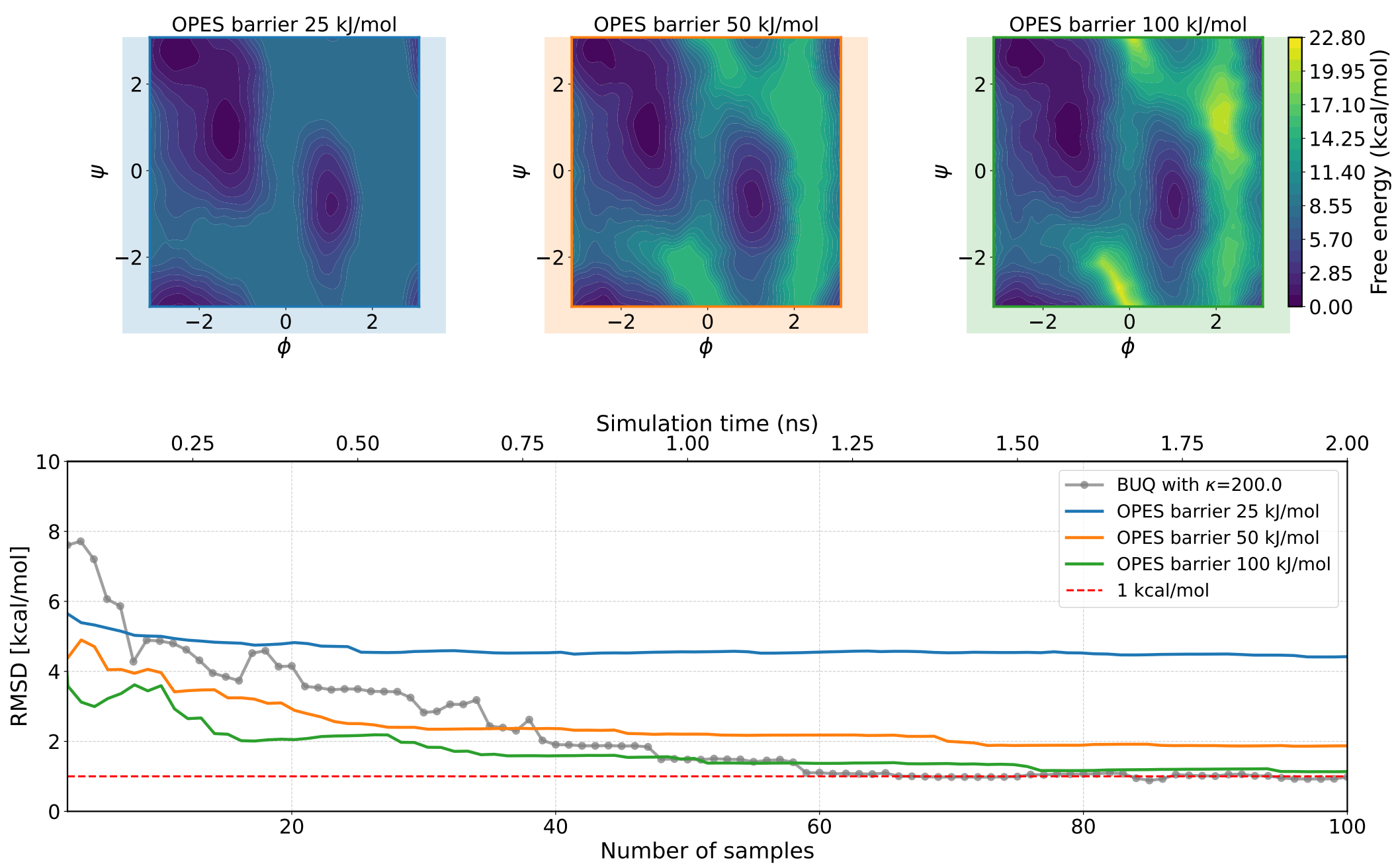

Figure S3: Comparison between OPES and BUQ for the reconstruction of the free-energy surface (FES) in the (ϕ, ψ) dihedral space of alanine dipeptide. (a–c) FES obtained from OPES simulations with barrier parameters of (a) 25 kJ/mol, (b) 50 kJ/mol, and (c) 100 kJ/mol, respectively. The color map indicates the free energy in kcal/mol. (d) Convergence of the RMSD (in kcal/mol) between each method's FES and a converged metadynamics reference

S.3.7 Effects of restraining parameter κ

We also investigated the sensitivity of BUQ to the restraint parameter, κ, used in the harmonic bias. In contrast to conventional umbrella sampling combined with WHAM, where κ controls window overlap and can strongly affect the reconstructed FES, BUQ-based umbrella integration is expected to be less sensitive to this choice, because it relies on mean forces rather than on histogram overlap.

Besides the default choice $\kappa = 200$, we considered $\kappa = 100$ (half) and $\kappa = 400$ (double), keeping all other hyperparameters identical. For each value of κ, we generated a BUQ free-energy surface (FES) in the (ϕ, ψ) dihedral space and monitored convergence using the same RMSD-based protocol as in earlier comparisons: the RMSD was computed with respect to the converged metadynamics reference as a function of the number of BUQ samples (equivalently, the effective simulation time up to 2 ns).

The resulting FES for $\kappa = 100, 200$, and 400 are visually very similar, indicating that BUQ is robust to these changes in the restraint strength. Quantitatively, all three choices of κ reach comparable final RMSD values within the 2 ns budget, with only minor differences in the early stages of sampling. For comparison, we also report the convergence curve for the best OPES setup (barrier 100 kJ/mol); BUQ attains RMSD values at or below the 1 kcal/mol threshold within a similar or smaller effective simulation time.

All Python scripts, PLUMED templates, and COLVAR files used to generate and post-process the BUQ training data (including the hyperparameter sweep) are provided in the repository. All restrained MD simulations were performed with GROMACS 2023 patched with PLUMED 2.9.1 using `UNITS LENGTH=A TIME=ps ENERGY=kcal/mol`.

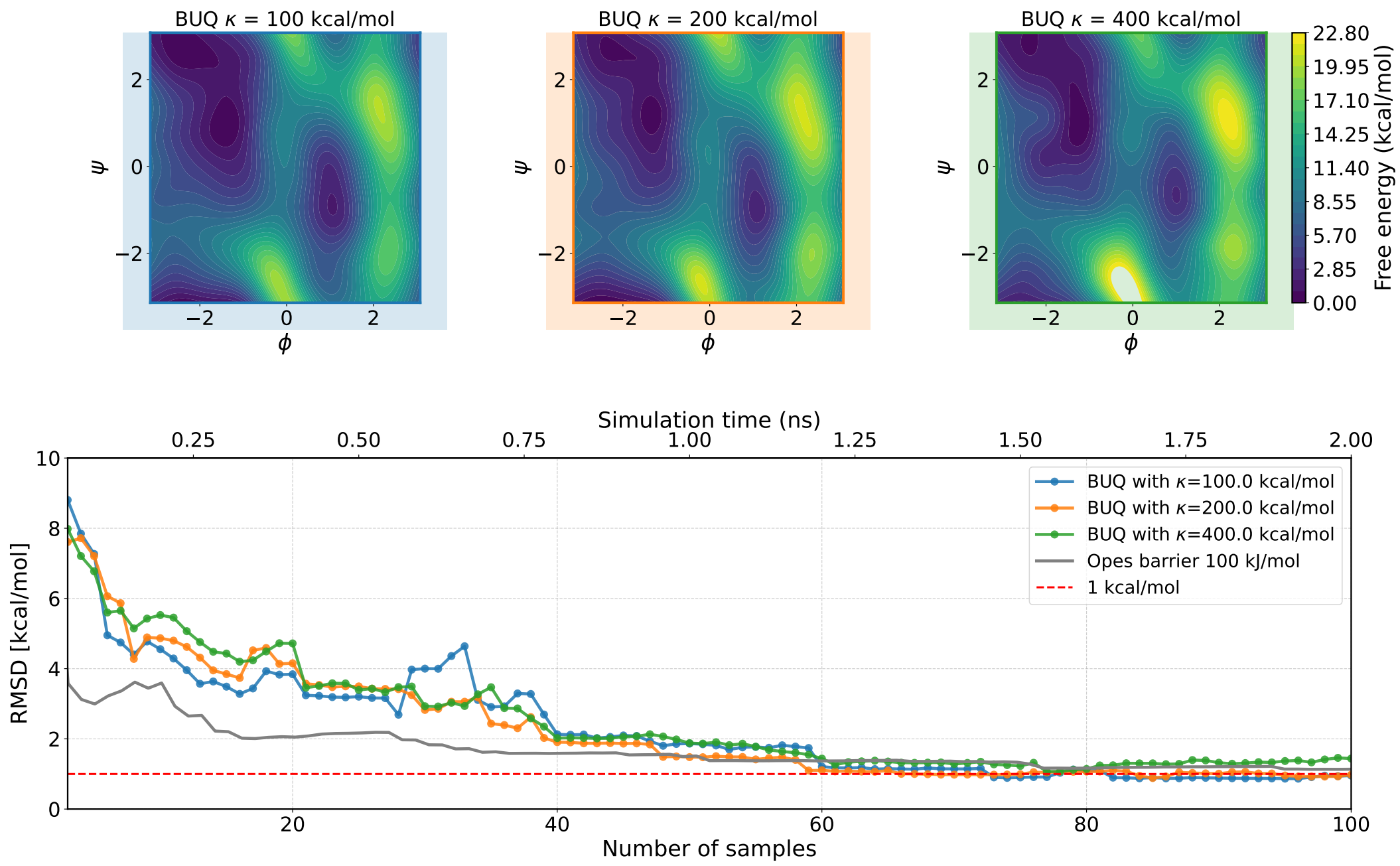

Figure S4: Effect of the BUQ restraint parameter κ on the reconstructed free-energy surface (FES) in the (ϕ, ψ) dihedral space. (a–c) BUQ FES obtained with (a) $\kappa = 100$, (b) $\kappa = 200$, and (c) $\kappa = 400$. The color map shows the free energy in kcal/mol and illustrates that all three restraint strengths produce very similar landscapes. (d) Convergence of the RMSD (in kcal/mol) with respect to a converged metadynamics reference as a function of the number of samples (bottom axis) and the corresponding simulation time (top axis). Shown are BUQ with $\kappa = 100$ (blue), $\kappa = 200$ (orange), and $\kappa = 400$ (green), together with the OPES run with barrier 100 kJ/mol (gray). The horizontal dashed line marks 1 kcal/mol. All BUQ variants reach comparable final RMSD values and approach or cross the 1 kcal/mol threshold within the considered simulation window, confirming the weak sensitivity of BUQ to the precise choice of the restraint strength κ.

S.4 Phase transition from water to ice

S.4.1 Molecular Dynamics protocol.

All simulations were carried out with LAMMPS (29 Aug 2024)[15] (units = `real`) patched with PLUMED 2.9.1,[11] using the TIP4P water model.[16] The simulation box contained 864 water molecules, initialized from an equilibrated ice configuration. MD simulations were performed with a 2 fs timestep using velocity rescaling[10] at 300 K with a 100 fs time constant, and an isotropic NPH barostat[17] at 1 bar with a 1 ps time constant, and SHAKE[18] constraints applied to O–H bonds and H–O–H angles. This setup and LAMMPS input files are taken from Ref.[19]

S.4.2 Umbrella Sampling protocol

The CV, the environment similarity order parameter,[20] of this simulation is defined as follows: Two environment similarities were defined, comparing instantaneous oxygen environments to reference ice structures (hexagonal and cubic). These descriptors yielded scalar, atom-specific order parameters q_{Ih} and q_{Ic}. To avoid competition between the two ice phases, we restrained the normalized growth of the system-averaged $\bar{q}_{\mathrm{Ic}}$ to be less than that of the target $\bar{q}_{\mathrm{Ih}}$. For more details, see.[19] In practice, we bias the number of Ih ice-like oxygens N_{ice}, with an q_{Ih} value higher than 0.5.

All queries started from an ice system, i.e., $N_{\mathrm{ice}} = 288$. The biasing schedule is similar to the alanine dipeptide case. First, the κ is gradually built up from 0 to 100 kJ per particle in 1000 timesteps. Then, it is moved to the desired window target in 49000 timesteps. We simulate for 15 ns in total, and we use the last 14 ns for statistics. CV values were stored every 500 steps. All PLUMED files are available in our repository.

S.4.3 Bayesian umbrella quadrature protocol

To initialize the simulations, we selected four initial samples near the two thermodynamically stable states along the order parameter N_{ice}. For the liquid basin, we used $N_{\text{ice}} = 0.6$ and 2.7. The lower value, $N_{\text{ice}} = 0.6$, corresponds to the point at which the histogram of N_{ice} obtained from unbiased MD trajectories in the liquid region decays to zero, while $N_{\text{ice}} = 2.7$ was chosen such that the most probable liquid value (the mode $N_{\text{ice}} \approx 1.7$) lies approximately in the middle of the interval, see Fig. S5. Analogously, for the crystalline basin we selected $N_{\text{ice}} = 282.6$ and 287.1: $N_{\text{ice}} = 287.1$ is located where the crystalline histogram vanishes, and $N_{\text{ice}} = 282.6$ was chosen so that the crystalline mode ($N_{\text{ice}} \approx 284.8$) is centered between the two crystalline starting points, see Fig. S6. These four values therefore bracket the liquid and solid free-energy minima and are determined from histogram analyses of unbiased MD simulations in the vicinity of each stable state.

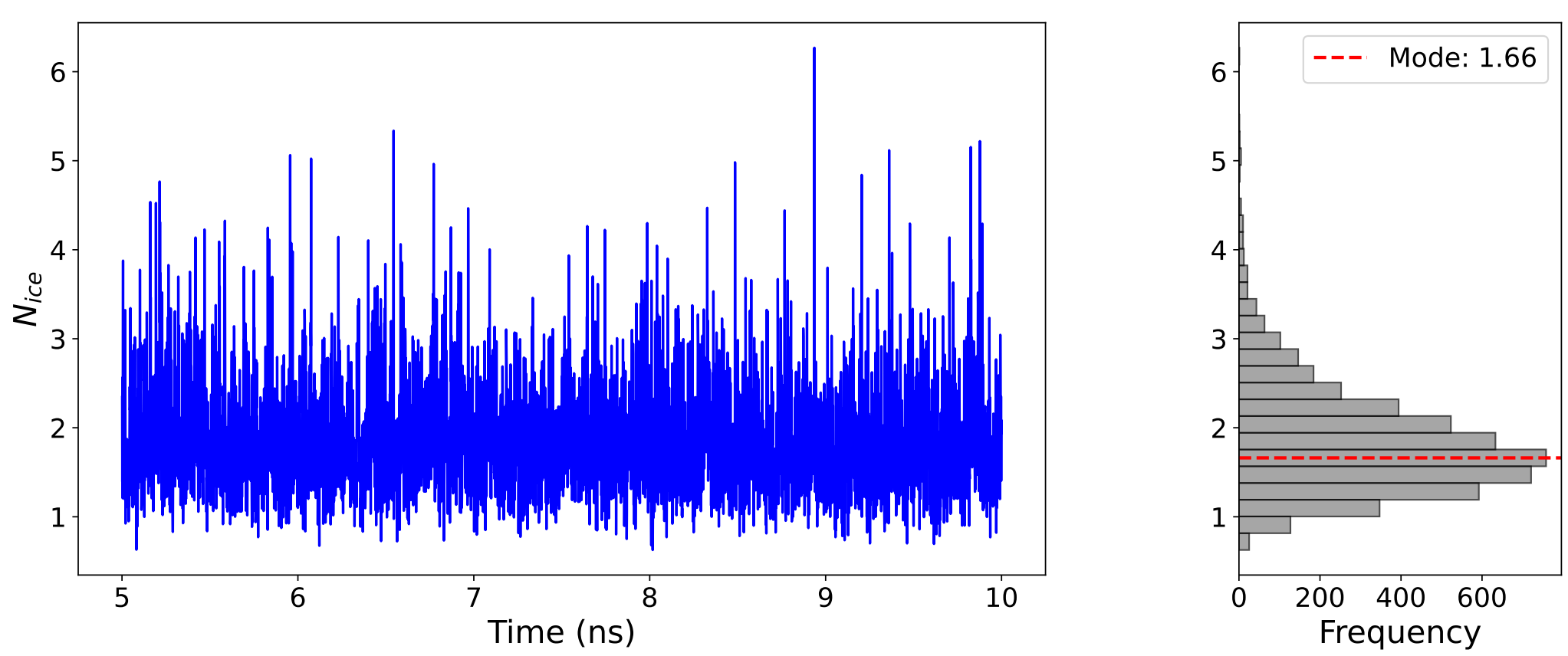

Figure S5: Histogram of the ice-order parameter N_{ice} from unbiased MD simulations in the liquid basin, used to select initial states near the liquid free-energy minimum. The values $N_{\text{ice}} = 0.6$ and 2.7 employed as starting points in the BUQ procedure are chosen such that the mode of the liquid distribution (at $N_{\text{ice}} \approx 1.66$) is bracketed by these two configurations and the lower value lies where the histogram vanishes.

After these four initial points, we use BUQ with IVR acquisition to obtain 15 more queries, resulting in 19 simulations in total. The baseline for accuracy is the reference free-energy profile reported in Ref.[19] The evolution of the root-mean-square deviation (RMSD)

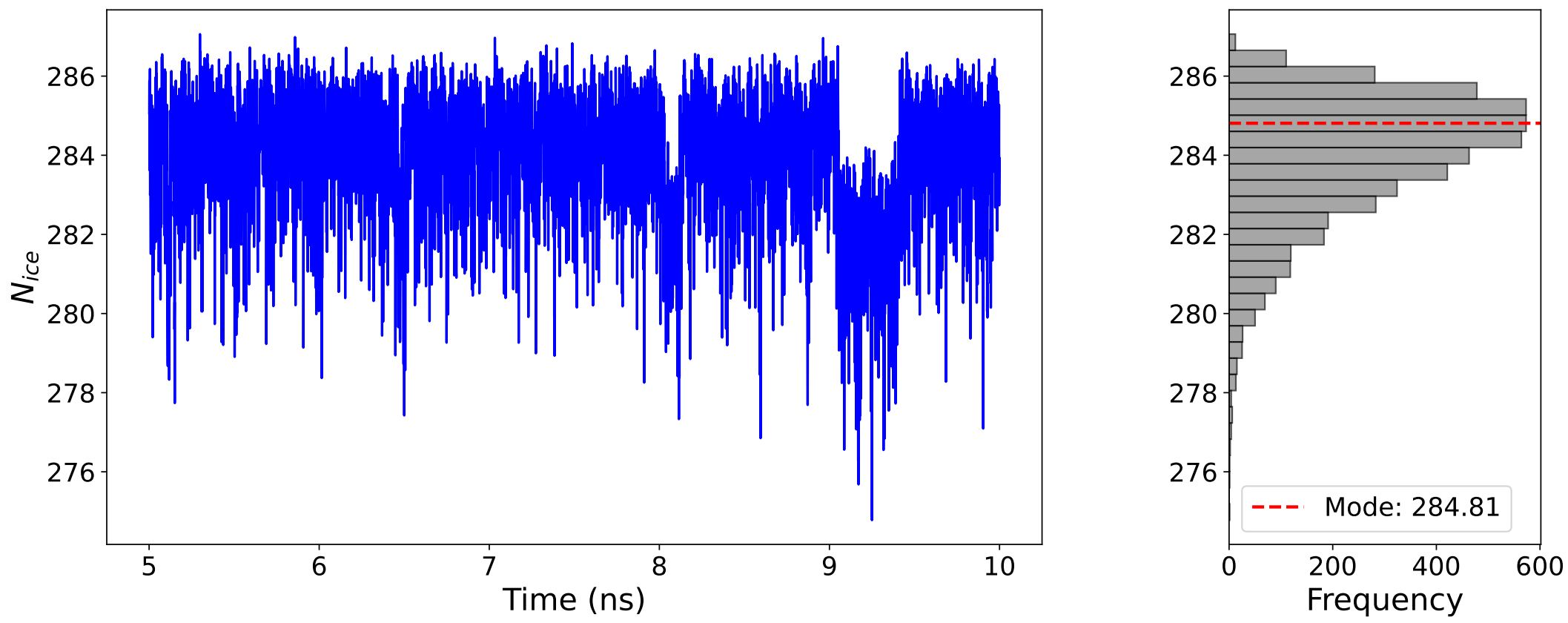

Figure S6: Histogram of the ice-order parameter N_{ice} from unbiased MD simulations in the crystalline basin, used to select initial states near the crystal free-energy minimum. The values $N_{\text{ice}} = 282.6$ and 287.1 are chosen such that the crystalline mode (at $N_{\text{ice}} \approx 284.8$) lies between them and the higher value lies where the histogram decays to zero.

with respect to this reference after each query is shown in Fig. S7.

Unlike the alanine-dipeptide system, the free-energy gradient of this phase transition is a more ragged and non-smooth function, reflecting nucleation and melting. To model this, we restricted the kernel family to Matérn-1/2 , which is able to capture sharp changes in the field, see also Fig. S8. The GP was wrapped in Emukit's quadrature interface with a Lebesgue prior over range of N_{ice}, (0, 288), trained on pairs $\{N_{\text{ice},i}\,, -F_i\}$, and integrated via trapezoidal rule to yield a free-energy profile, shown in the main text.

We tested a small hyperparameter grid. Force constant κ_{ice} was fixed at 100 kJ per particle throughout production runs. An adaptive-κ variant was evaluated, using a higher κ in regions where a steeper derivative was predicted. However, for this system we did not see an effect, perhaps because the base κ value was already strong enough to drive nucleation. The kernel type was Matérn-1/2, known to handle non-smoothness more effectively than RBF, with lengthscales $\ell \in \{10, 20\}$ N_{ice}. The Gaussian white-noise variance was set to zero because additional noise obscured the steep gradients near the stable states, leading the GP to misinterpret them as stochastic fluctuations. Query budgets were $N_{\text{queries}} = 15$, with and without the exploitation acquisition term $\lambda \in \{0, 0.1\}$.

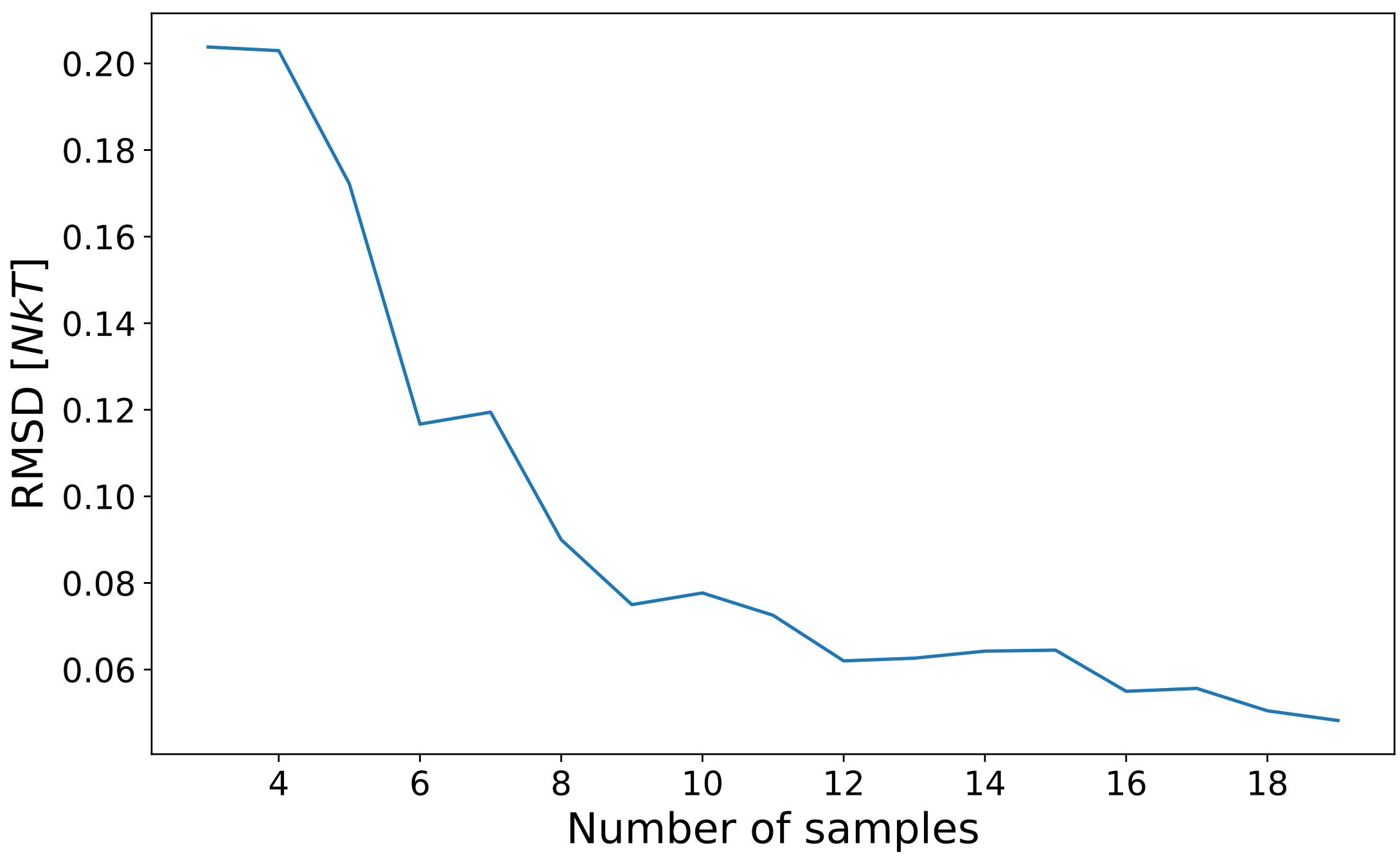

Figure S7: Root-mean-square deviation (RMSD) of the estimated free-energy profile with respect to the reference profile of Ref.,[19] as a function of the number of BUQ queries for the ice order parameter N_{ice}. [2]

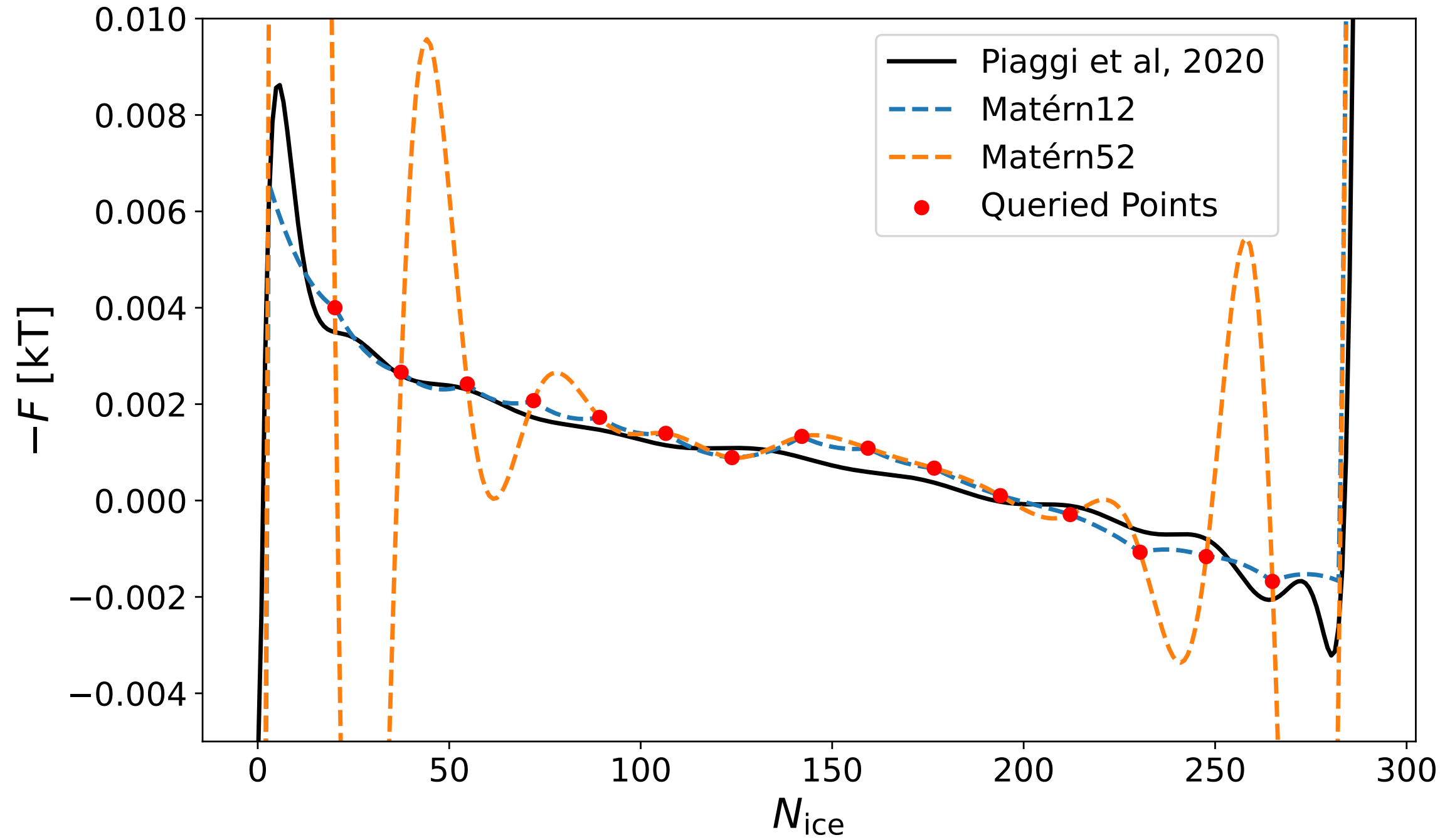

Figure S8: Free-energy derivative $-F$ as a function of N_{ice} for the ice–liquid phase transition. The black solid line shows the reference profile from Ref.,[19] while the dashed curves correspond to Gaussian process reconstructions with Matérn-1/2 and Matérn-5/2 kernels. Red indicates the 19 queried simulation points obtained through BUQ with IVR acquisition, starting from four initial samples near the stable liquid and crystal states. The Matérn-1/2 kernel successfully captures the ragged, non-smooth structure of the free-energy gradient associated with nucleation and melting.

After testing, a lengthscale of 20 N_{ice} and $\lambda \in \{0.1\}$ delivered the best performance. However, we note that in some runs we experienced outliers around $N_{\text{ice}} \approx 175$. This was caused by the forming of an ice cylinder across the periodic boundaries of the box, which can be easily solved by using a elongated box. Moreover, outliers can be easily corrected within the BUQ framework, only a simple refitting of the GP is required.

S.5 $\mathbf{S_N2}$ chemical reaction between ethyl chloride and a fluoride anion

S.5.1 Molecular dynamics protocol

We use a machine-learned reactive potential reported in Ref.[21] The MD simulations were carried out with ASE,[22] and used a 0.5 fs timestep with a velocity rescaling thermostat at 300 K .[10]

S.5.2 Unbiased barrier crossings from the reactant state

We performed a set of short, essentially unbiased MD simulations to characterize the stable states and the dynamical barrier crossing. In these runs, the system was initialized in the reactant basin and propagated with the same ML potential and thermostat as described above for a total of 100 ps per trajectory. The collective variables d_1 and d_2 were monitored but not restrained by harmonic biases; instead, we only applied soft upper and lower walls at $d_1 \in [1.2, 2.8]$ Å and $d_2 \in [1.8, 3.5]$ Å to prevent the dynamics from exploring unphysical regions of configuration space. Angular restraints on the reacting moiety were kept as in the umbrella simulations to avoid rotations that would lead outside the training domain of the ML potential.

Figure S9 shows the resulting trajectories in the (d_2, d_1) plane from 12 independent runs, color-coded by simulation time. The two minima corresponding to the reactant and product

states are clearly visible, and all trajectories cross the barrier between them within the 100 ps simulation window. These spontaneous barrier crossings are consistent with the very low free-energy barrier obtained from the BUQ and umbrella-integration free-energy surfaces. However, they do not provide enough statistics to accurately compute the free energy barrier.

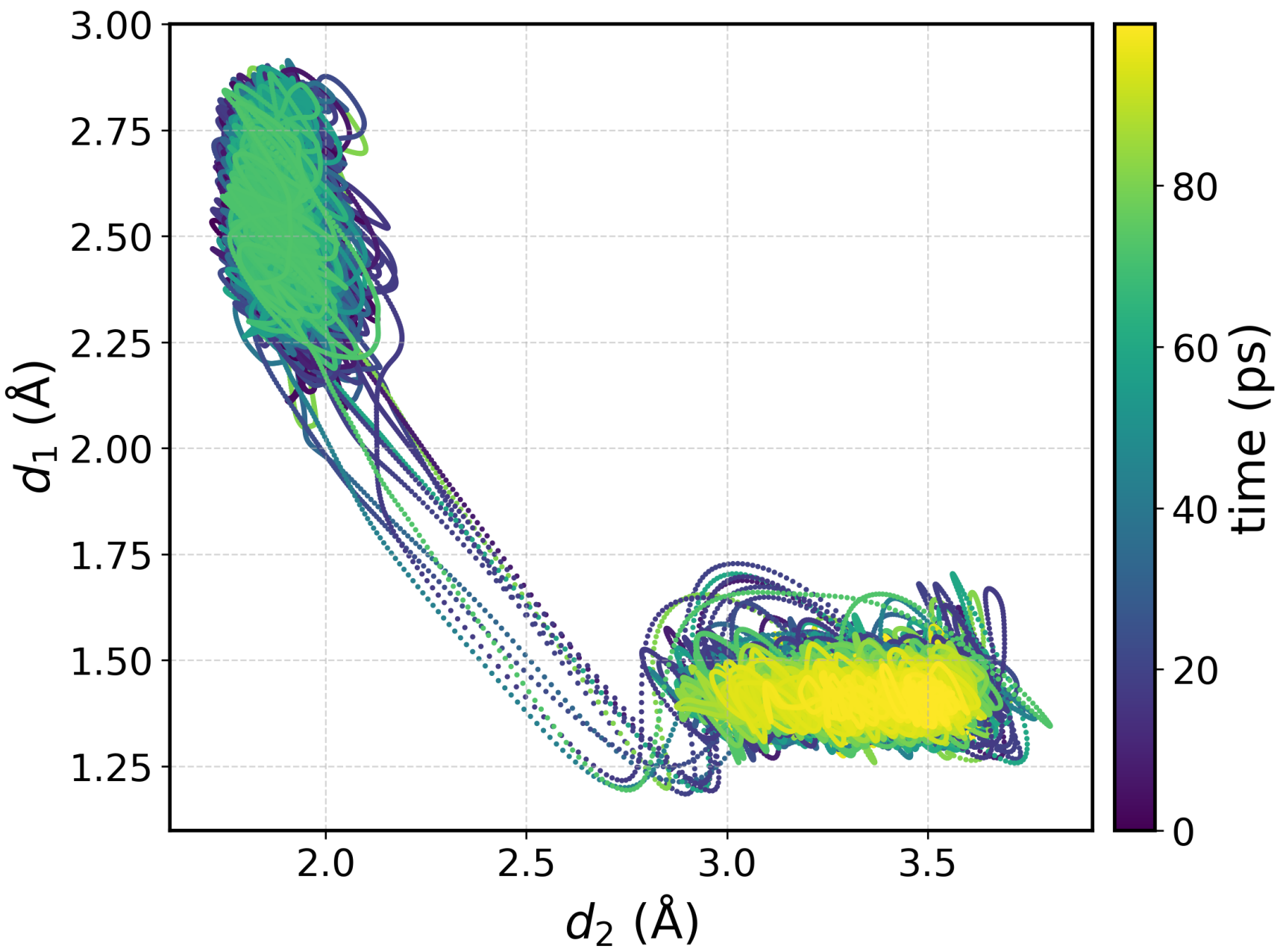

Figure S9: Unbiased trajectories of the collective variables describing the S_N2 reaction. Each point shows (d_2, d_1) from 12 independent 100 ps MD trajectories of the ethyl chloride + F^- system, propagated with the ML potential at 300 K. The color scale indicates the simulation time in ps. The two basins correspond to the reactant state (C–Cl bound, large d_1 and small d_2) and the product state (C–F bound, small d_1 and large d_2). Apart from soft upper and lower walls at $d_1 \in [1.2, 2.8]$ Å and $d_2 \in [1.8, 3.5]$ Å and angular restraints on the reacting fragment, no bias was applied to d_1 or d_2. The frequent transitions between the two basins within 100 ps are consistent with a very low free-energy barrier for this reaction.

S.5.3 Umbrella sampling protocol

All simulations were initialized with the ethyl initially bound to the chloride ion (the reactant). We describe the reaction with two CVs, d_1 and d_2, i.e., the distances from the ethyl carbon to the fluorine and chlorine atoms, respectively. Restraints were applied through PLUMED to enforce the desired geometries: the distances d_1 and d_2 were restrained with moving harmonic restraints that gradually steered the system from the starting geometry

$(2.64, 1.84)$ Å to the target window (d_1, d_2). The pulling along a reaction coordinate of the form $r_c = d_2 - d_1$ was avoided due to the difference in strength of the C–F bond and C-Cl bond. We used a $\kappa_{d_1} = 1000$kcal/(mol× Å) at 2.64 and a $\kappa_{d_2} = 100$kcal/(mol × Å) at 1.84, which were both built up gradually in 1000 timesteps. Then, we used 4000 timesteps to move the restraint to the desired d_1 and d_2. Angular restraints were applied to prevent rotations of the reactive moiety, which could take the system into untrained configurations for the ML potential. CV values were stored every 100 steps. Each simulation ran for 40 ps, sufficient to equilibrate around the target geometry and compute average biasing forces on d_1 and d_2.

S.5.4 Bayesian umbrella quadrature protocol

We considered the ranges $d_1 \in [1.2, 2.8]$ Å and $d_2 \in [1.8, 3.5]$ Å. We used the The Matérn-5/2 kernel with a lengthscale of 0.2 Å in both dimensions. We set $\lambda = 0.2$ for exploiting the minima, and we set the noise to 0. We used three initial samples: two at the minima and one along the path connecting the minima, with values $(d_1, d_2) =$ (2.64, 1.84), (1.6, 2.6), (1.413, 3.468). Then, we used the IVR acquisition to obtain 50 more samples, so 53 in total. During the analysis of the trajectories, we identified two BUQ umbrellas in which a hydrogen atom escaped into the gas phase. These umbrellas, originally centered at $(d_1, d_2) =$ (2.28, 1.94) and (2.50, 3.24), were discarded and replaced by the closest available samples from the UI benchmark, $(d_1, d_2) =$ (2.27, 1.99) and (2.44, 3.31), respectively. The original (red squares) and replacement (red circles) positions are highlighted in Fig. S11.

Finally, the BUQ-predicted free-energy surface was interpolated onto a 60×60 grid, and a minimum free-energy path (MFEP) was extracted using the string method.[23] To obtain a benchmark, we carried out conventional two-dimensional umbrella integration (UI) along d_1 and d_2 on an 11×11 grid. The d_2 values were $d_2 \in \{1.80, 1.89, 1.99, 2.08, 2.18, 2.37, 2.56, 2.74, 2.93, 3.12, 3.31\}$ Å, and the d_1 values were $d_1 \in \{1.20, 1.38, 1.56, 1.73, 1.91, 2.09, 2.27, 2.36, 2.44, 2.62, 2.80\}$ Å, chosen such that the grid is denser near the reactant minimum,

allowing for improved sampling in that region. For the UI data, we reconstructed the FES using the same GP setup as for BUQ (Matérn-5/2 kernel, length scale 0.2 Å in both directions), see S11. Here, we excluded one umbrella window in which the hydrogen atom dissociated, in order to avoid contamination of the UI reference surface by unphysical dissociated configurations, namely $(d_1, d_2) = (2.27, 3.12)$. To assess the convergence of BUQ, we computed the root-mean-square deviation (RMSD) between the BUQ free-energy surface and the final UI benchmark after each additional BUQ sample; see Fig. S10. The final RMSD is 0.07286 eV, i.e., 1.680 kcal/mol.

To find the MFEP on the BUQ surface, we first initialized a straight-line path between the reactant (C–Cl bound) and product (C–F bound) states, discretized into 20 nodes. Using the string method,[23,24] the path was then relaxed using a fourth-order spline representation, with fixed endpoints, for a maximum of 150 iterations, saving the path every 10 steps. The converged string reproduces the geometry reported in Ref.[21] Next, we used this converged BUQ string as the initial guess for a refined string on the UI benchmark FES and the BUQ FES, with a larger number of nodes (200), and relaxed it for 3 additional iterations to allow for some adaptation to the landscape while retaining comparable paths. We then projected the free energy along this final paths for both BUQ and UI. The resulting profiles exhibit very similar shapes. The corresponding reaction barriers are 0.0053 eV for the BUQ fes, and 0.0075 eV for the UI benchmark, showing good mutual consistency, also shown in S11. Both barriers are smaller than the barrier on the potential-energy surface reported in Ref.,[21] which is expected for a free-energy profile due to entropic contributions. Consistently, the valleys in the free-energy landscape are broader than in the potential-energy surface, reflecting these entropic effects.

As an additional benchmark for the S_N2 reaction, we explored different OPES (on-the-fly probability enhanced sampling) settings. We varied the OPES pace, the initial configuration (reactant or product) and we did try the employed the OPES `explore` protocol as well. The barrier parameter was set to 200 kJ/mol, $\approx$ 2 eV. Among these tests, we also applied

the same upper and lower walls on d_1 and d_2 (PLUMED `UPPER_WALLS` and `LOWER_WALLS`) as in the unbiased simulations in Sec. S.5.2, since without these soft boundaries the OPES dynamics would likely drive the system outside the physically meaningful (d_1, d_2) region. All these simulations were performed on the Snellius supercomputer (SURF, The Netherlands) using a single GPU node. Jobs were submitted via `SLURM` with a typical allocation of one NVIDIA H100 GPU and 32 CPU cores per run. Each simulation was run for up to 5 days of wall-clock time on Snellius (the default limit), corresponding to typical total simulation times of ~ 0.7 ns. However, the resulting free-energy surfaces showed noticeable dependence on the initial configuration and OPES settings, and convergence was not unequivocal. Among the tested setups, OPES-`explore` yielded the most reasonable FES; these results are reported in Fig. S12. Moreover, when analyzing the trajectories from OPES explore, in all cases bonds not involved in the reaction were broken, including the bonds with hydrogen atoms and the C–C bond (see Fig. S12). The earliest of these breakages occurs at 4 ps. This again indicates likely underlying issues when running MD with this Machine Learning Potential (MLIP). Nonetheless, discrepancies with the UI benchmark remain, as do differences between simulations started from reactant versus product configurations. In addition, applying the string method to the OPES-derived FES did not lead to a stable and converged path in this case.

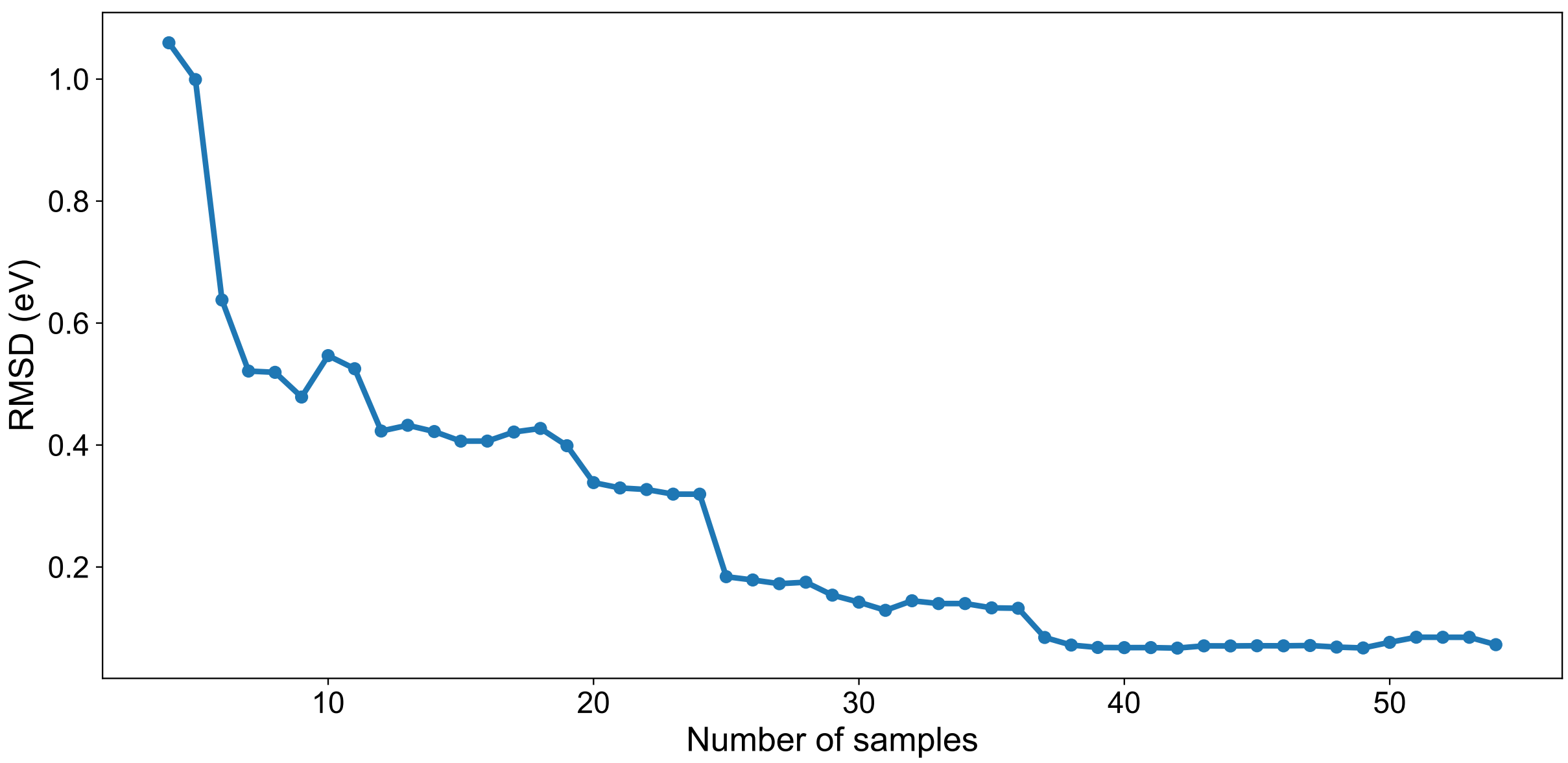

Figure S10: Root-mean-square deviation (RMSD) of the BUQ free-energy surface with respect to the final UI benchmark after each additional BUQ sample.

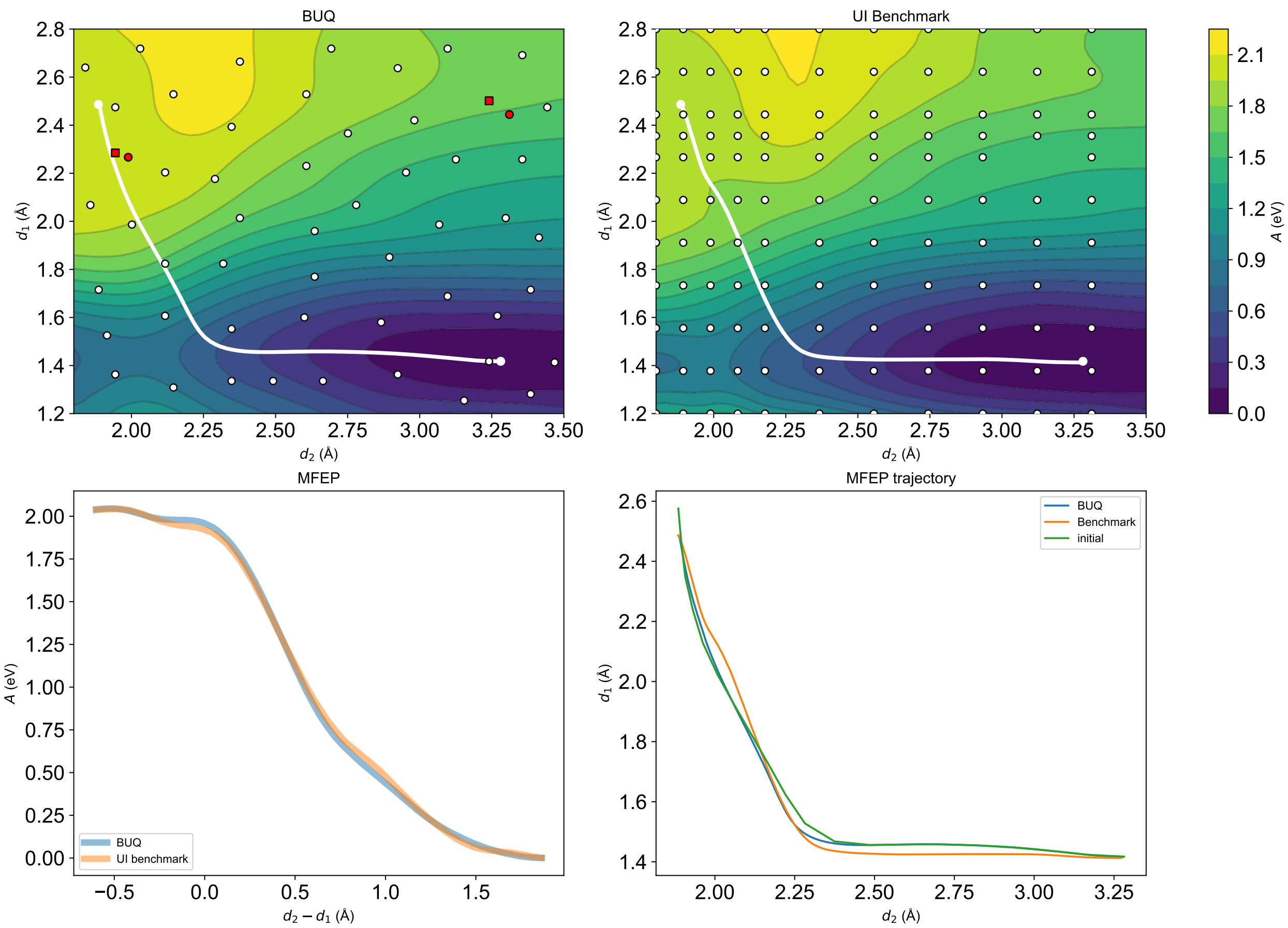

Figure S11: Comparison between BUQ and umbrella-integration (UI) free-energy surfaces (FES) and minimum free-energy paths (MFEPs) for the S_N2 reaction in the (d_2, d_1) collective-variable space, where d_1 and d_2 are the C–F and C–Cl distances, respectively. (a)BUQ FES (color map, in eV) with BUQ sampling locations (white circles) and the converged MFEP obtained by the string method (white line). The two red squares indicate the original BUQ umbrellas in which hydrogen atoms escaped into the gas phase, and the nearby red circles mark the replacement samples taken at the closest umbrella centers from the UI benchmark. (b) UI benchmark FES reconstructed from the 11×11 umbrella grid, with window centers (white circles) and the corresponding MFEP (white line). (c) One-dimensional free-energy profiles $A(r_c)$ along the MFEPs, projected onto $d_2 - d_1$, comparing BUQ (blue) and UI benchmark (orange); the two profiles exhibit nearly identical shapes and barrier heights. (d) MFEP trajectories in (d_2, d_1) space: initial path (green), BUQ-optimized path (blue), and UI-refined benchmark path (orange), illustrating the close agreement between the BUQ and UI transition pathways.

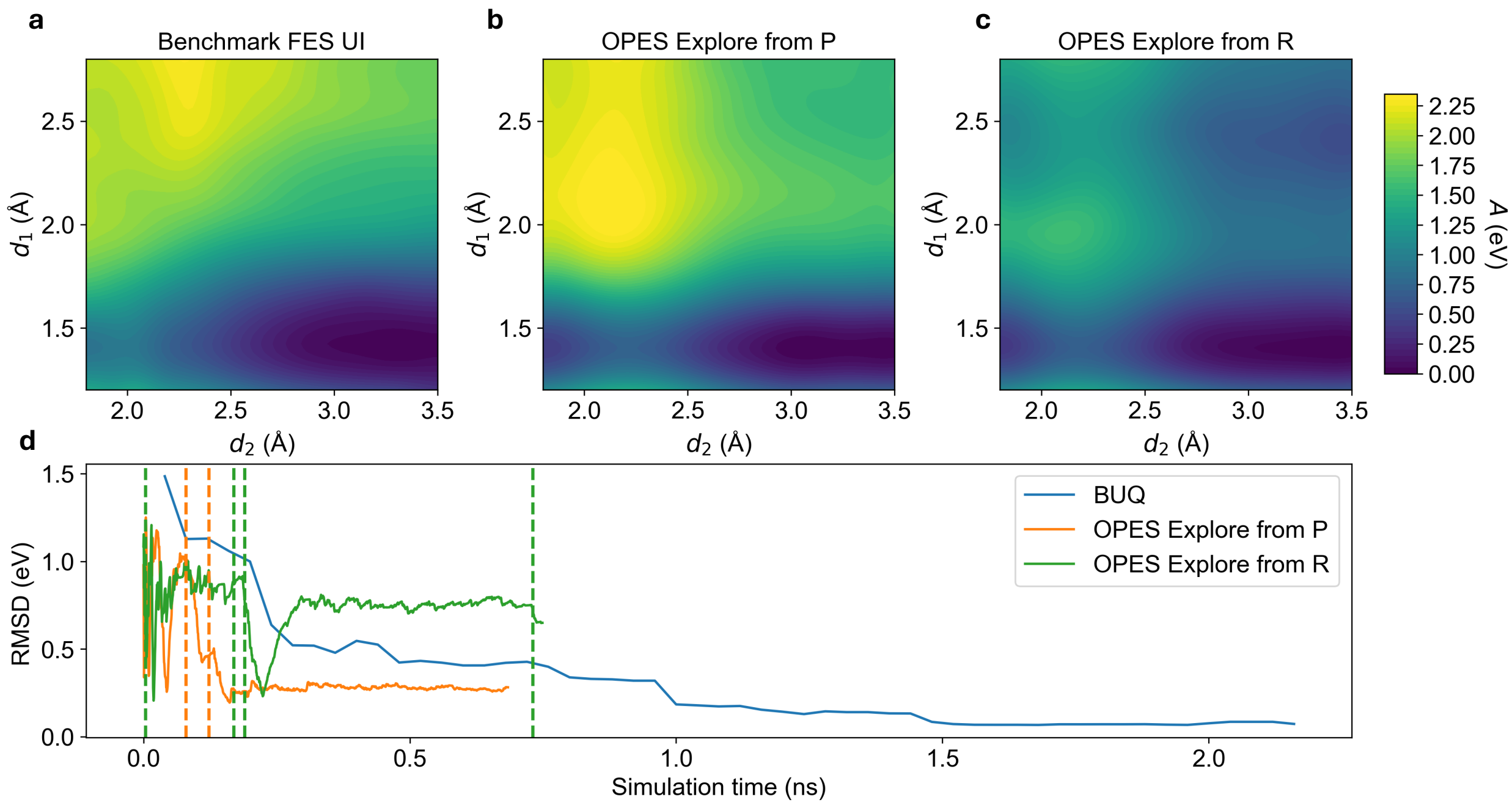

Figure S12: Comparison between umbrella-integration (UI) and OPES-`explore` free-energy surfaces (FES) and their convergence for the S_N2 reaction. (a) Benchmark FES obtained from two-dimensional umbrella integration in the (d_2, d_1) space, where d_1 and d_2 are the C–F and C–Cl distances, respectively. (b) FES reconstructed from an OPES-`explore` simulation started from the product (P) state. (c) FES reconstructed from an OPES-`explore` simulation started from the reactant (R) state. (d) RMSD convergence analysis between the OPES-derived FES and the UI benchmark, for simulations started from P (blue) and R (orange). The vertical lines indicate the times when bonds not involved in the reaction were broken.

Code and Data availability

Code to reproduce all analysis and results is available at:

`https://github.com/computational-chemistry-uva/BUQ`.

References

(1) Rasmussen, C. E.; Williams, C. K. I. *Gaussian processes for machine learning*; MIT Press, 2006; p 248.

(2) GPy GPy: A Gaussian process framework in python. 2012.

(3) Paleyes, A.; Pullin, M.; Mahsereci, M.; McCollum, C.; Lawrence, N.; González, J. Emulation of physical processes with Emukit. Second Workshop on Machine Learning and the Physical Sciences, NeurIPS. 2019.

(4) Paleyes, A.; Mahsereci, M.; Lawrence, N. D. Emukit: A Python toolkit for decision making under uncertainty. *Proceedings of the Python in Science Conference* **2023**,

(5) Gessner, A.; Gonzalez, J.; Mahsereci, M. Active Multi-Information Source Bayesian Quadrature. Proceedings of The 35th Uncertainty in Artificial Intelligence Conference. 2020; pp 712–721.

(6) Gunter, T.; Osborne, M. A.; Garnett, R.; Hennig, P.; Roberts, S. J. Sampling for Inference in Probabilistic Models with Fast Bayesian Quadrature. Advances in Neural Information Processing Systems. 2014.

(7) Berendsen, H. J. C.; van der Spoel, D.; van Drunen, R. GROMACS: A message-passing parallel molecular dynamics implementation. *Computer Physics Communications* **1995**, *91*, 43–56.

(8) Lindorff-Larsen, K.; Piana, S.; Palmo, K.; Maragakis, P.; Klepeis, J. L.; Dror, R. O.; Shaw, D. E. Improved side-chain torsion potentials for the Amber ff99SB protein force field. *Proteins: Structure, Function, and Bioinformatics* **2010**, *78*, 1950–1958.

(9) Hess, B.; Bekker, H.; Berendsen, H. J. C.; Fraaije, J. G. E. M. LINCS: A linear constraint solver for molecular simulations. *Journal of Computational Chemistry* **1997**, *18*, 1463–1472.

(10) Bussi, G.; Donadio, D.; Parrinello, M. Canonical sampling through velocity rescaling. *The Journal of Chemical Physics* **2007**, *126*, 014101.

(11) Tribello, G. A.; Bonomi, M.; Branduardi, D.; Camilloni, C.; Bussi, G. PLUMED 2: New feathers for an old bird. *Computer Physics Communications* **2014**, *185*, 604–613.

(12) Promoting transparency and reproducibility in enhanced molecular simulations. *Nature Methods* **2019**, *16*, 670–673.

(13) Boneta, S. Umbrella Integrator. `https://github.com/boneta/UmbrellaIntegrator`, 2021.

(14) Invernizzi, M.; Parrinello, M. Rethinking Metadynamics: From Bias Potentials to Probability Distributions. *The Journal of Physical Chemistry Letters* **2020**, *11*, 2731–2736, PMID: 32191470.

(15) Thompson, A. P.; Aktulga, H. M.; Berger, R.; Bolintineanu, D. S.; Brown, W. M.; Crozier, P. S.; in 't Veld, P. J.; Kohlmeyer, A.; Moore, S. G.; Nguyen, T. D.; Shan, R.; Stevens, M. J.; Tranchida, J.; Trott, C.; Plimpton, S. J. LAMMPS - a flexible simulation tool for particle-based materials modeling at the atomic, meso, and continuum scales. *Computer Physics Communications* **2022**, *271*, 108171.

(16) Abascal, J. L.; Vega, C. A general purpose model for the condensed phases of water: TIP4P/2005. *The Journal of Chemical Physics* **2005**, *123*.

(17) Shinoda, W.; Shiga, M.; Mikami, M. Rapid estimation of elastic constants by molecular dynamics simulation under constant stress. *Physical Review B* **2004**, *69*, 134103.

(18) Kräutler, V.; van Gunsteren, W. F.; Hünenberger, P. H. A fast SHAKE algorithm to solve distance constraint equations for small molecules in molecular dynamics simulations. *Journal of Computational Chemistry* **2001**, *22*, 501–508.

(19) Piaggi, P. M.; Car, R. Phase equilibrium of liquid water and hexagonal ice from enhanced sampling molecular dynamics simulations. *The Journal of chemical physics* **2020**, *152*, 204116.

(20) Piaggi, P. M.; Parrinello, M. Calculation of phase diagrams in the multithermal-multibaric ensemble. *The Journal of Chemical Physics* **2019**, *150*.

(21) Kuryla, D.; Csányi, G.; van Duin, A. C.; Michaelides, A. Efficient exploration of reaction pathways using reaction databases and active learning. *The Journal of Chemical Physics* **2025**, *162*.

(22) Hjorth Larsen, A. et al. The atomic simulation environment—a Python library for working with atoms. 2017; `https://doi.org/10.1088/1361-648X/aa680e`.

(23) E, W.; Ren, W.; Vanden-Eijnden, E. Simplified and improved string method for computing the minimum energy paths in barrier-crossing events. *The Journal of Chemical Physics* **2007**, *126*, 164103.

(24) E, W.; Ren, W.; Vanden-Eijnden, E. String method for the study of rare events. *Physical Review B* **2002**, *66*, 52301.
